# Natriuretic Peptide‐Guided Therapy in Acute Decompensated Heart Failure: An Updated Systematic Review and Meta‐Analysis

**DOI:** 10.1002/clc.70165

**Published:** 2025-06-04

**Authors:** Luciana Gioli‐Pereira, Eric Shih Katsuyama, Christian Ken Fukunaga, Wilson Falco, Camila Campos Grisa Padovese, Rafael Hortencio Melo, Edielle de Sant'Anna Melo, Silvana E. Ribeiro Papp, Fernando Bacal

**Affiliations:** ^1^ Department of Cardiology and Intensive Care Hospital Municipal Dr Gilson de Cássia Marques de Carvalho, Sociedade Beneficente Israelita Brasileira Albert Einstein São Paulo Brazil; ^2^ Faculdade Israelita de Ciências da Saúde Albert Einstein Hospital Israelita Albert Einstein São Paulo São Paulo Brazil; ^3^ Department of Medicine Faculty of Medicine of ABC Santo André Brazil; ^4^ Department of Medicine Faculdade de Medicina de Catanduva Catanduva Brazil; ^5^ Department of Medicine Universidade Estadual de Londrina Londrina Paraná Brazil; ^6^ Department of Cardiology, Hospital Municipal Dr Gilson de Cássia Marques de Carvalho Sociedade Beneficente Israelita Brasileira Albert Einstein São Paulo Brazil; ^7^ UPMC Harrisburg Pennsylvania USA; ^8^ Department of Cardiology Sociedade Beneficente Israelita Brasileira Albert Einstein São Paulo Brazil; ^9^ Instituto do Coração (InCor) São Paulo Brazil

**Keywords:** heart failure, hospitalization, mortality, natriuretic peptides, usual care

## Abstract

**Background:**

Natriuretic peptides (NP) are widely used to diagnose heart failure (HF), but their role in guiding treatment remains uncertain. We performed a randomized trial meta‐analysis comparing NP‐guided therapy to usual care in acute decompensated HF.

**Methods:**

We searched PubMed, Embase, and Cochrane for RCTs comparing NP‐guided therapy to usual care in acute decompensated HF. Outcomes included all‐cause mortality, cardiovascular death, and a composite of mortality and HF hospitalizations (reported as RR and 95% CI). Heterogeneity was assessed using *I* ^2^, and a random‐effects model was applied when appropriate. Analyses were performed in R Studio 4.3.2.

**Results:**

We included 9 RCTs with 3992 patients, of whom 2007 (50.3%) underwent NP‐guided treatment. The median follow‐up was 12 months. All‐cause mortality (RR: 0.84; 95% CI: 0.69–1.01; *p* = 0.069; *I* ^2^ = 41%), cardiovascular death (RR: 0.91; 95% CI: 0.78–1.08; *p* = 0.287; *I* ^2^ = 0%), and the composite outcome of HF hospitalization or cardiovascular death (RR: 0.91; 95% CI: 0.77–1.09; *p* = 0.308; *I* ^2^ = 56%) were not significantly different between groups. The time to event analysis of all‐cause mortality had a slightly significant advantage in favor of NP‐guided therapy (HR: 0.81; 95% CI: 0.69–0.95; *p* = 0.01; *I* ^2^ = 0%).

**Conclusion:**

Although NP‐guided therapy showed a statistically significant benefit in time to all‐cause mortality, this was not consistently reflected across other endpoints, and its overall clinical impact remains uncertain.

## Graphical abstract

Natriuretic peptides can help diagnose acute heart failure, but their role in treatment remains unclear. A meta‐analysis of 9 RCTs (*N* = 3992) found NP‐guided therapy slightly reduced time to all‐cause mortality but did not significantly impact overall mortality, cardiovascular death, or hospitalizations, suggesting limited benefit in clinical outcomes.

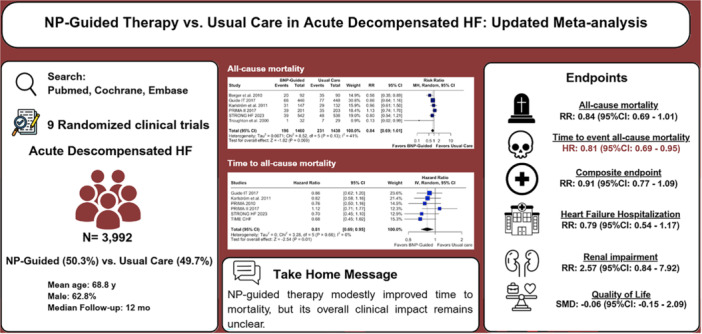

**Abbreviations**
ACC/AHA: American College of Cardiology, American Heart Association
ADHF: acute decompensated heart failure
BNP: B‐type natriuretic peptide
CI: confidence interval
CV: cardiovascular
GDMT: guideline‐directed medical therapy
HF: heart failure
HR: hazard ratio
NP: natriuretic peptide
NT‐proBNP: N‐terminal pro‐BNP
OR: odds ratio
PRISMA: Preferred Reporting Items for Systematic Reviews and Meta‐Analysis
RCT: randomized controlled trial(s)
RIS: required information size
RR: risk ratio
SMD: standard mean difference

## 1 Introduction

Heart failure (HF) is a complex syndrome with high mortality and hospitalization rates [1]. The management of HF is generally based on signs and symptoms (conventional or usual care), but medication adjustments can also be guided by biomarkers, specifically natriuretic peptides (NPs)—a strategy known as “NP‐guided therapy” [2].

NPs, such as B‐type natriuretic peptide (BNP) and N‐terminal pro‐BNP (NT‐proBNP), are invaluable for diagnosing and prognosticating HF. They are potent predictors of mortality and HF hospitalization, irrespective of ejection fraction and HF etiology. BNP is an active neurohormone that counteracts the renin–angiotensin–aldosterone system and sympathetic nervous system overactivity in HF, while NT‐proBNP is an inactive prohormone released from cardiomyocytes under wall stress [3].

However, despite the strong prognostic value of NPs, there is insufficient evidence and a lack of consensus on the routine use of serial BNP and NT‐proBNP measurements for guiding HF therapy or optimizing guideline‐directed medical therapy (GDMT) in chronic HF. Randomized controlled trials (RCTs) and meta‐analyses have produced conflicting results regarding the direct benefits of NP‐guided HF treatment on mortality and hospitalization rates [4]. Acute decompensated heart failure (ADHF) is a high‐risk condition requiring prompt treatment. Due to rapid volume changes and elevated event rates, NP‐guided strategies may be especially useful in this setting. Considering this controversy, we performed an updated meta‐analysis of RCTs evaluating the NP‐guided therapy compared to usual care in patients with ADHF.

## 2 Methods

This systematic review and meta‐analysis were performed and reported under the Cochrane Collaboration Handbook for Systematic Reviews of Interventions and the Preferred Reporting Items for Systematic Reviews and Meta‐Analysis (PRISMA) statement guidelines [5, 6] and are depicted in Methods S1 and S2. The protocol was published in the International Prospective Register of Systematic Reviews, PROSPERO (CRD42024579101).

### 2.1 Eligibility Criteria

Inclusion was restricted to studies that met all the following eligibility criteria: (1) RCTs; (2) comparing NP‐guided therapy and usual care; (3) enrolling patients with ADHF; and (4) with no restrictions on follow‐up time. For this study, ADHF was defined as a sudden worsening of HF symptoms requiring urgent medical intervention, regardless of whether it represented a de novo presentation or an exacerbation of chronic HF. This definition included patients admitted for ADHF as well as those recently discharged following a hospitalization for the condition. In addition, studies were included only if they reported any of the clinical outcomes of interest. We excluded studies (1) with no control group; (2) outpatients; (3) overlapping study population; (4) without reports on any of the outcomes of interest; and (5) abstracts from congresses. There was no restriction regarding the publication date, status, or language.

### 2.2 Search Strategy and Data Extraction

We systematically searched PubMed, Embase, and Cochrane Central Register of Controlled Trials from inception to May 2024 with the following search terms: “natriuretic peptide‐guided treatment,” “biomarker‐guided therapy,” “usual care,” “symptom‐guided treatment,” “acutely decompensated HF,” and “hospital setting.” The complete search strategy is available in Methods S3. The study selection process included reviewing titles and abstracts initially, followed by a thorough examination of the full texts of potentially suitable studies. The references from all included studies, previous systematic reviews, and meta‐analyses were also searched manually for any additional studies.

We collected baseline characteristics for: (1) study name and year; (2) follow‐up time; (3) age; (4) female population; (5) baseline NT‐proBNP levels; (6) mean left ventricular ejection fraction at baseline; (7) number of patients in NYHA I, II, III, and IV; (8) creatinine levels at baseline; (9) country; (10) drug used in the usual care; (11) HF type; and (12) type of blinding. We also collected data for the selected outcomes, eligibility criteria per study, definition of composite outcome per study, and target and titration criteria per study. Five authors (R.H.M., C.C.G.P., E.S.A.M., C.K.F., and W.F.) independently and following a double‐blinded model, extracted selected studies, reviewed the main reports and supporting materials, and extracted the relevant information from the included trials. Any discrepancies were resolved through consensus among the authors or addressed through deliberation with other review team members (L.G.P. and E.S.K.).

### 2.3 Endpoints and Subgroup Analysis

The primary endpoint was all‐cause mortality. Secondary outcomes included (2) composite outcomes; (3) cardiovascular (CV) death; (4) HF hospitalizations; (5) quality of life; and (6) adverse events, specifically (i) hypotension and (ii) renal impairment. We performed a subgroup analysis for time to composite outcome according to age groups under and over 75 years old. Detailed definitions of each endpoint are provided in Methods S4.

### 2.4 Quality Assessment

Two review authors (E. S. A. M. and E. S. K.) independently assessed the risk of bias for each trial using the criteria outlined in the *Cochrane Handbook for Systematic Reviews of Interventions*, through Cochrane's Risk of Bias 2 tool for randomized studies according to the following domains: random sequence generation, allocation concealment, blinding of participants and personnel, blinding of outcome assessment, incomplete outcome data, selective outcome reporting, and other biases [6, 7]. We resolved disagreements by discussion or by a third review author (L. G. P.). We graded each trial as having a high, low, or unclear risk of bias for each domain. Publication bias was investigated by funnel‐plot analysis of point estimates according to study weights and by Egger's regression test.

### 2.5 Statistical Analysis

Endpoints were analyzed using a risk ratio (RR) with 95% confidence intervals (CIs). We also computed hazard ratio (HR) with 95% CIs for a time‐to‐event analysis. Heterogeneity was examined with the *I* ^2^ statistic. Continuous means were reported using standard mean difference (SMD) with 95% CIs following an effect size model. We used a random‐effect model for outcomes with low heterogeneity (< 25%). R (R Foundation for Statistical Computing, Vienna, Austria) version 4.3.2 was used for statistical analyses using the “meta” package. Description of Kaplan–Meier curve extraction strategy is detailed in Methods S5. We performed leave‐one‐out and Baujat plot methods to address heterogeneity and sensitivity analysis, which are provided in Methods S6.

## 3 Results

### 3.1 Study Selection and Baseline Characteristics

**Figure 1:**
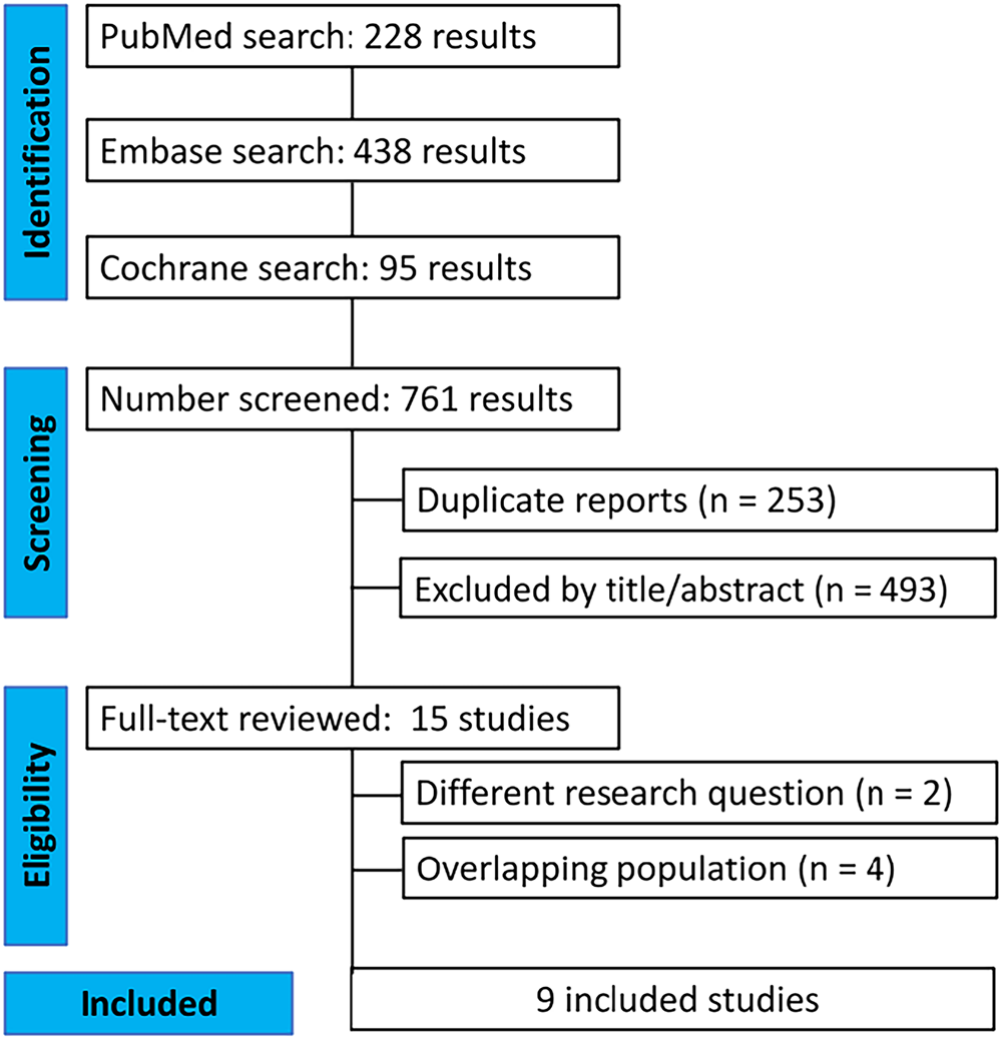
PRISMA flow diagram of study screening and selection.

The study selection is demonstrated in Figure 1. The initial search identified 761 studies (PubMed [*n* = 228], Embase [*n* = 438], and Cochrane [*n* = 95]). After title and abstract screening and removal of duplicates, 15 studies remained to be fully reviewed according to the inclusion and exclusion criteria. Of these, 9 RCTs were included, comprising 3992 patients, of whom 2007 (50.29%) were treated according to NT‐proBNP guided [8, 9, 10, 11, 12, 13, 14, 15, 16]. According to the eligibility criteria, six studies were excluded: one due to addressing a different research question, four due to overlapping populations, and one for not reporting the outcomes of interest. A full description of the eligibility criteria per study can be found in Table S1. The included subjects had a mean age of 68.79 years and were mostly male (62.8%). The follow‐up ranged between 4 and 24 months, with a median of 12 months. Study characteristics are in Table 1 and Table S2. Additionally, each study's composite outcome definition and target BNP for optimization criteria for the NP‐guided group can be found in Tables S3 and S4, respectively.

**Table 1:** Baseline characteristics of included studies.

Study	FU (mo)	Patients (*n*)		Age (y)		Female (%)		NT‐proBNP (pg/mL)		LVEF (%)		NYHA I, II		NYHA III, IV		Cr (mg/dL)	
		NP	UC	NP	UC	NP	UC	NP	UC	NP	UC	NP	UC	NP	UC	NP	UC
Berger et al. [10]	12	92	90	70 ± 12	71 ± 13	40	34	2216 [355–9649]^c^	2359 [355–15 603]^c^	NA	NA	NA	NA	NA	NA	NA	NA
GUIDED‐IT [22]	24	446	448	62 [51–70]^c^	64 [52–72]^c^	31	33	2632 [1462–5235]^c^	2668 [1481–5604]^c^	24 [19–30]^c^	25 [20–30]^c^	254 (58%)	252 (57%)	184 (42%)	191 (43%)	1.3 [1.1–1.7]^c^	1.3 [1.1–1.7]^c^
Karlstrom [13]^a^	12	147	132	72 ± 9.7^b^	70 ± 10^b^	27	27	808.2 ± 676.1^b^	898.9 ± 915.3^b^	NA	NA	47 (32%)	36 (27%)	98 (67%)	96 (73%)	1.2 ± 0.4^b^	1.2 ± 0.4^b^
Lainchbury [16]	36	121	121	76 [44–89]^c^	76 [34–89]^c^	37	33	2012 [516–10 233]^c^	1996 [425–6588]^c^	40 ± 15^b^	39 ± 15^b^	80 (66%)	73 (60%)	20 (16%)	27 (22%)	1.4 ± 0.5^b^	1.4 ± 0.6^b^
PRIMA [14]	24	174	171	72 ± 12^b^	73 ± 12^b^	45	40	8034 [4210–13 831]^c^	8168 [4288–14 051]^c^	35 ± 14^b^	37 ± 15^b^	133 (76%)	128 (80.7%)	41 (23.6%)	33 (19.3%)	1.4 [1.1–1.8]^c^	1.4 [1.2–1.9]^c^
PRIMA II [9]	6	201	203	78 [69–85]^c^	77 [68–84]^c^	54	48	6293 [3949–11 438]^c^	6122 [3252‐11 371]^c^	36 ± 15^b^	38 ± 15^b^	37 (18%)	44 (22%)	159 (79%)	151 (74%)	1.3 [1–1.8]^c^	1.2 [0.9–1.7]^c^
STRONG‐ HF [12]	6	542	536	63 ± 13.5^b^	63 ± 13.7^b^	40	37	4121 ± 3677^b^	3929 ± 3213^b^	37 ± 13^b^	36 ± 12^b^	176 (35%)	194 (38%)	332 (67%)	298 (60%)	NA	NA
TIME‐CHF [15]	18	252	248	76 ± 7^c^	77 ± 8^c^	32	37	3998 [2075–7220]^c^	4657 [2455–7520]^c^	30 ± 8^b^	30 ± 8^b^	65 (25.9%)	63 (25.4%)	186 (74.1%)	185 (74.6%)	1.3 ± 0.4^b^	1.3 ± 0.4^b^
Troughton et al. [11]	6	33	36	68	72	22	25	4774	5522	28	26	72	67	28	33	NA	NA

*Note:* Binary data reported in number (proportion).

Abbreviations: Cr = creatinine, FU = follow‐up, LVEF = left ventricular ejection fraction, NA = not available, NP = natriuretic peptide‐guided treatment, NYHA = New York Heart Association, UC = usual care.

a BNP level.

^b^ Mean ± SD.

^c^ Median [IQR].

### 3.2 Pooled Analysis for Efficacy Endpoints

**Figure 2:**
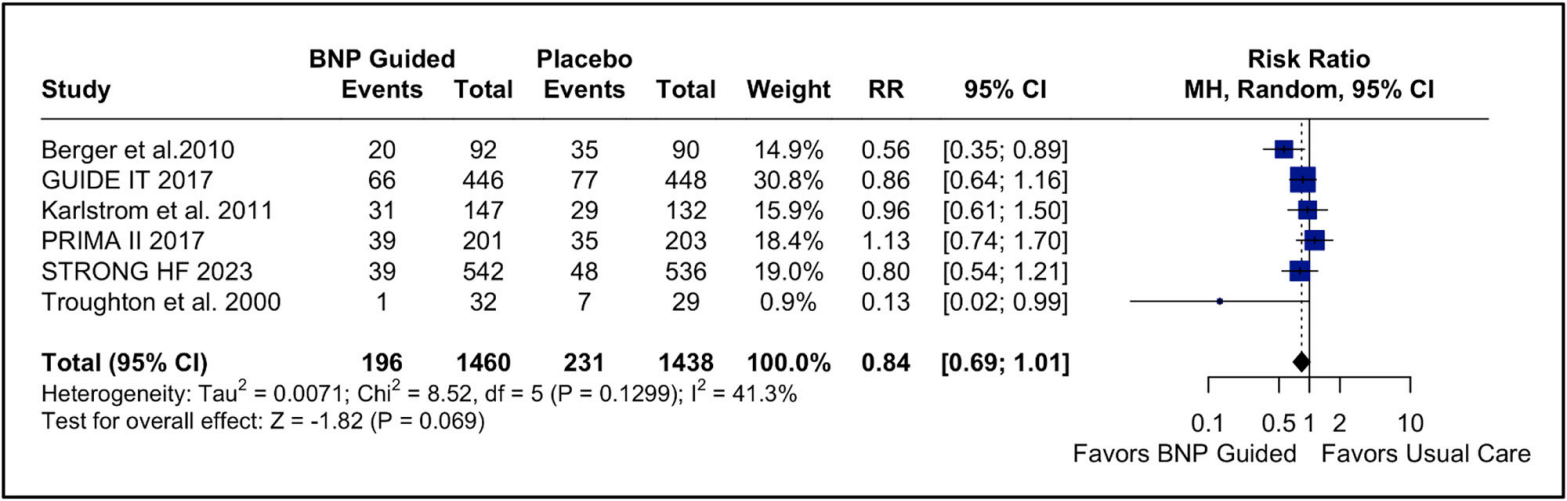
Forest plot for all‐cause mortality. In patients with acute decompensated heart failure, BNP‐guided therapy was not significantly associated with a change in risk of all‐cause mortality endpoint compared to usual care. BNP, brain natriuretic peptide; CI, confidence interval; MH, Mantel–Haenszel; RR, risk ratio.

**Figure 3:**
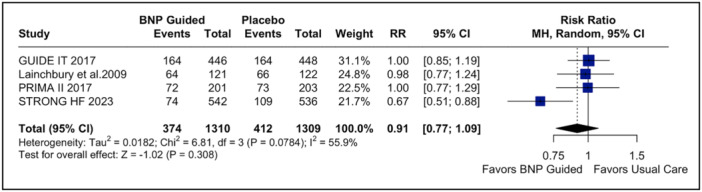
Forest plot for the composite endpoint. In patients with acute decompensated heart failure, BNP‐guided therapy was not significantly associated with a change in risk of composite endpoint compared to usual care. BNP, brain natriuretic peptide; CI, confidence interval; MH, Mantel–Haenszel; RR, risk ratio.

**Figure 4:**
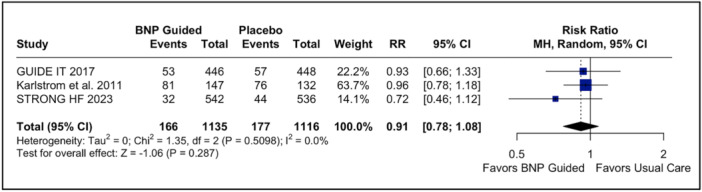
Forest plot cardiovascular death endpoint. In patients with acute decompensated heart failure, BNP‐guided therapy was not significantly associated with cardiovascular death endpoint compared to usual care. BNP, brain natriuretic peptide; CI, confidence interval; IV, inverse variance.

Our pooled analysis for the primary endpoint of all‐cause mortality showed no difference in NP‐guided treatment compared to usual care (RR: 0.84; 95% CI: 0.69–1.01; *p* = 0.069; *I* ^2^ = 41%; Figure 2). The time to event all‐cause mortality had a significant advantage in favor of NP‐guided (HR: 0.81; 95% CI: 0.69–0.95; *p* = 0.01; *I* ^2^ = 0%; Figure S1). The composite outcome demonstrated no statistically significant difference between the group receiving NP‐guided treatment and the usual care group (RR: 0.91; 95% CI: 0.77–1.09; *p* = 0.308; *I* ^2^ = 56%; Figure 3), consistent with the time‐to‐event analysis (HR: 0.80; 95% CI: 0.63–1.02; *p* = 0.07; Figure S2). In addition, CV death (RR: 0.91; 95% CI 0.78–1.08; *p* = 0.287; *I* ^2^ = 0%; Figure 4) and its time‐to‐event analysis revealed no statistically significant difference between the NP‐guided treatment group and the usual care group (RR: 0.94; 95% CI: 0.74–1.19; *p* = 0.58; *I* ^2^ = 0%; Figure S3). The analysis of HF hospitalization revealed no statistically significant change between the NP‐guided treatment group and the usual care group (RR: 0.79; 95% CI: 0.54–1.17; *p* = 0.241; *I* ^2^ = 88%; Figure S4).

### 3.3 Quality of Life

**Figure 5:**
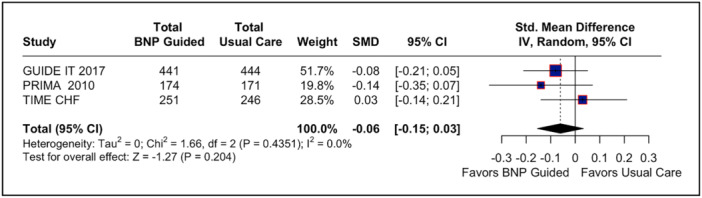
Forest plot for quality‐of‐life endpoint. In patients with acute decompensated heart failure, BNP‐guided therapy was not significantly associated with quality‐of‐life improvement endpoint compared to usual care. BNP, brain natriuretic peptide; CI, confidence interval; IV, inverse variance; MH, Mantel–Haenszel; Std., standard.

The pooled analysis of quality‐of‐life outcomes, analyzed in three studies, showed no statistically significant difference between the NP‐guided treatment group and the usual care group (SMD: −0.06; 95% CI −0.15 to 2.09; *p* = 0.40; *I* ^2^ = 83%; Figure 5).

### 3.4 Pooled Analysis for Safety Endpoints

There was no significant difference between groups in the incidence of hypotension analyzed in four studies (RR: 2.94; 95% CI 0.81–10.61; *p* = 0.1; *I* ^2^ = 68%; Figure S5A). Similarly, the incidence of renal impairment, analyzed in three studies, was not significantly different between groups (RR: 2.57; 95% CI: 0.84–7.92; *p* = 0.099; *I* ^2^ = 61%; Figure S5B).

### 3.5 Subgroup Analysis

**Figure 6:**
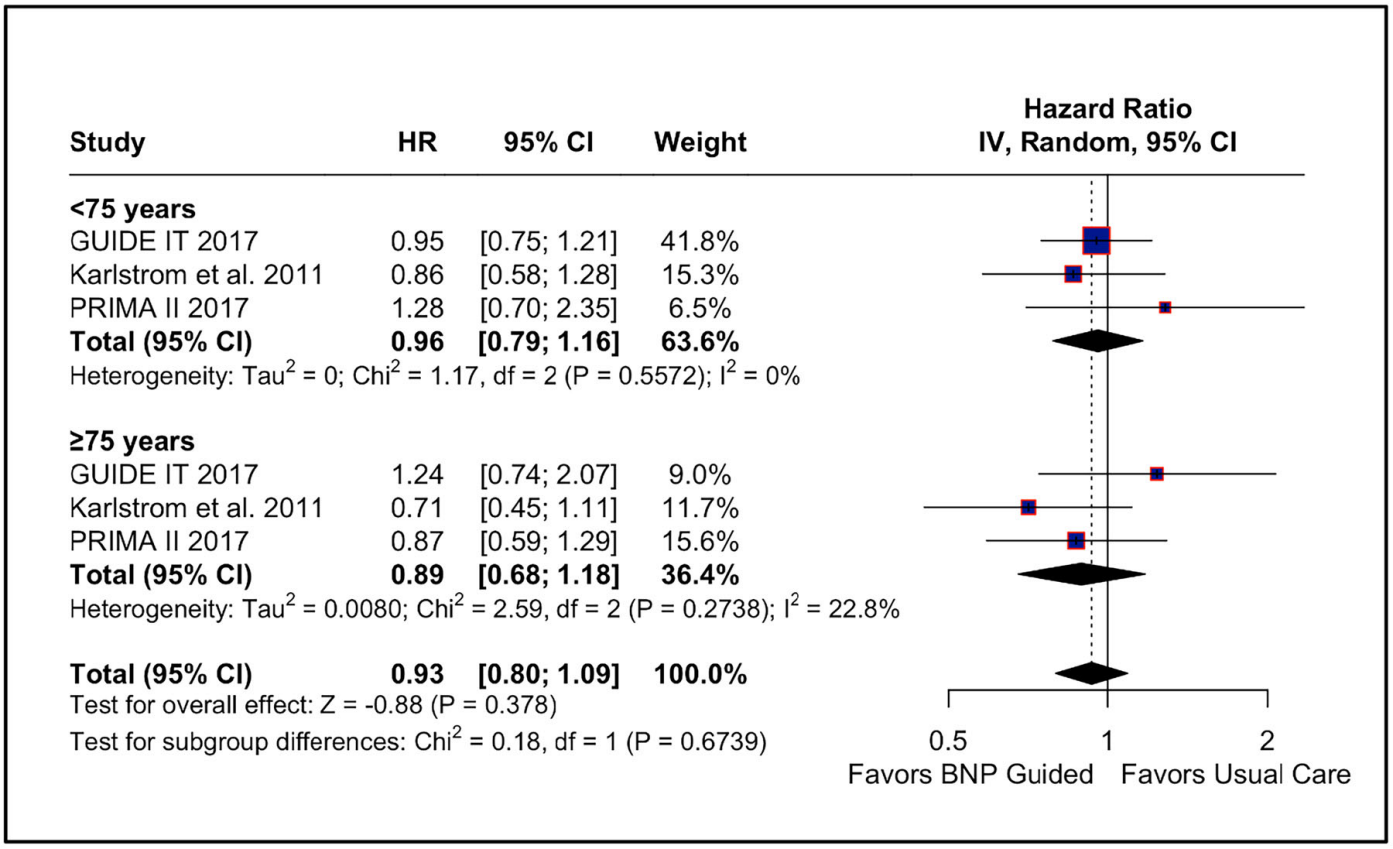
Forest plot for subgroup analysis for the composite endpoint. In our subgroup analysis, there was no significant subgroup association among patients with > 75 years old versus < 75 years old. BNP, brain natriuretic peptide; CI, confidence interval; MH, Mantel–Haenszel; RR, risk ratio.

We performed a subgroup analysis for time to composite outcome according to age groups. No statistical difference was observed in patients under 75 years (HR: 0.96; 95% CI: 0.79–1.16; *p* = 0.67; *I* ^2^ = 0%; Figure 6) and over 75 years (HR: 0.89; 95% CI: 0.68–1.18; *p* = 0.42; *I* ^2^ = 23%; Figure 6). Besides, no difference was found between the age groups (*p* = 0.67).

### 3.6 Sensitivity Analysis and Addressing Heterogeneity

Our sensitivity analysis using the leave‐one‐out and the Baujat methods for the all‐cause mortality endpoint suggests that the PRIMA II trial [9], Berger et al. trial [10], and Troughton et al. [11] are the potential outliers for this endpoint. After omitting Berger et al. [10] (RR: 0.90; 95% CI: 0.75–1.09; *I* ^2^ = 23%; Figure S6) and Troughton et al. [11] (RR: 0.85; 95% CI: 0.71–1.03; *I* ^2^ = 24%; Figure S6A,B), respectively, there was still no significant difference in all‐cause mortality between NP‐guided and usual care groups. However, after omitting the PRIMA II trial, NP‐guided therapy was associated with a significant risk reduction compared to the usual care group (RR: 0.79; 95% CI: 0.65–0.96; *I* ^2^ = 35%; Figure S6A,B).

### 3.7 Trial Sequential Analysis for the Primary Endpoint

A trial sequential analysis (TSA) was conducted to support our findings (Figure S7). For all‐cause mortality, the *Z*‐curve did not reach the required information size (RIS) and did not cross the significance threshold boundary, suggesting no difference between the NP‐guided and usual care groups. However, this result may be subject to a Type II error, indicating that more patients may be needed to meet the RIS and provide more robust conclusions.

### 3.8 Quality Assessment

Individual RCTs appraisal using the RoB 2 tool is depicted in Figure S8. Four studies were deemed low risk of bias, and five with moderate risk due to bias due to deviations from intended interventions. Funnel plot shows a symmetrical distribution of similar weight studies, suggesting no small study effect (Figure S9).

## 4 Discussion

We conducted a systematic review and meta‐analysis of 9 RCTs involving 3992 patients with decompensated HF, comparing NP‐guided therapy with standard care. Our main findings indicate no statistically significant differences between the NP‐guided therapy group and the usual care group in terms of all‐cause mortality, CV death, HF‐related death, composite outcomes, adverse effects, or quality of life.

NPs play a critical role in the diagnosis and prognosis of HF. These biomarkers are powerful predictors of mortality and HF‐related hospitalizations, regardless of ejection fraction or the underlying cause of HF. Despite their strong prognostic value, there is limited evidence and ongoing debate regarding the routine use of serial BNP and NT‐proBNP measurements to guide acute HF therapy versus standard care [17].

In the early 2000s, Troughton and colleagues provided the first evidence that NP‐guided therapy could improve outcomes in HF patients compared to standard clinically guided therapy [11]. Since then, numerous studies have produced conflicting results [18, 19]. McLellan and colleagues conducted a meta‐analysis with 4063 patients with HF, summarizing the available evidence and concluding in favor of NP‐guided therapy over standard care [20]. However, our updated systematic review and meta‐analysis, including the STRONG‐HF trial [12], suggests no significant advantage of NP‐guided therapy. The variability in HF populations across studies, characterized by different NYHA stages and both preserved and reduced left ventricular ejection fraction, likely contributes to these discrepancies and impacts the generalizability of the findings. HF admissions and all‐cause mortality are primary outcomes that we aimed to improve with GDMT. However, our pooled analysis revealed no significant difference compared to usual care.

Regarding HF hospitalization, there was no significant difference between NP‐guided and usual care, and high heterogeneity was observed. Similarly, there was no significant difference in all‐cause mortality between the two groups. High heterogeneity was present initially, but it was reduced when performing a leave‐one‐out analysis, specifically after removing studies by Berger and Troughton, resulting in *I* ^2^ values of 21% and 24%, respectively. Some possible explanations for this divergence could be differences in the study populations (e.g., HF, severity, age, comorbidities) that may influence the results. In this way, Berger and Troughton were the only studies that did not include a specific threshold for NT‐BNP in their inclusion criteria and had a small sample size among the included studies [10, 11].

In our meta‐analysis, we identified a statistically significant difference in time to all‐cause mortality, favoring NP‐guided therapy (HR 0.81; 95% CI 0.69–0.95; *p* = 0.01), with no observed heterogeneity (*I* ^2^ = 0%). This was the only outcome showing a clear benefit of NP‐guided therapy over usual care. Conversely, we did not observe significant differences between NP‐guided therapy and usual care regarding time to CV death or the composite outcomes of HF hospitalization and major CV events. The lack of statistical significance in these composite time‐to‐event outcomes may reflect the complexity of such endpoints, in which NP‐guided strategies may not exert a uniform effect across all individual components. These findings also underscore the importance of an analytical approach: while RR‐based comparisons yielded neutral results for all‐cause mortality, the time‐dependent analysis demonstrated a consistent benefit. Therefore, incorporating time‐to‐event methodologies is crucial when assessing the long‐term impact of biomarker‐guided interventions in HF management.

The observed trend toward benefit suggests that NP‐guided therapy may have clinical relevance, supporting its use in guiding HF treatment. However, one of the key challenges in interpreting the findings of this meta‐analysis is the heterogeneity in how NP levels were used to guide therapy across the included studies. While some trials implemented structured, protocol‐driven adjustments, others allowed clinician discretion, leading to variability in management strategies [8, 9]. This inconsistency may have influenced the results, potentially reducing the ability to detect the benefits of NP‐guided therapy. Moreover, the absence of a standardized protocol complicates direct comparisons between studies. Our findings underscore the need for future clinical trials with standardized protocols, where NP levels are used in a structured and reproducible manner, allowing for a more robust assessment of their impact on clinical outcomes. Additionally, future studies should provide detailed reporting of treatment interventions, ensuring a more precise analysis of therapeutic strategies.

For the remaining outcomes analyzed—CV death, quality of life, and composite outcomes—there were no significant differences between NP‐guided therapy and usual care. Regarding quality of life, the heterogeneity was initially high, but it reduced to 0% when the STRONG‐HF trial was excluded, indicating that this study was a major source of variability. In contrast, for the composite outcome, high heterogeneity persisted even after the leave‐one‐out analysis, suggesting that variability in results could not be attributed to any single study alone. We also performed a subgroup analysis based on age, using a cutoff of 75 years. This analysis revealed no significant differences between groups, indicating that age did not modify the effect of NP‐guided therapy on the outcomes assessed.

Finally, for adverse effects such as renal impairment and hypotension, there were no significant differences between NP‐guided therapy and usual care. However, the heterogeneity was initially high for both outcomes. Notably, when the STRONG‐HF trial was excluded, the heterogeneity for renal impairment reduced to 0%, again highlighting the impact of this study on overall variability.

Most randomized controlled trials conducted to date have shown no significant differences between NP‐guided therapy and standard care in HF management [2, 9, 13, 14, 15, 21]. We speculate that this discrepancy could be due to several factors. First, variations in inclusion criteria, such as differences in HF severity, patient age, comorbidities, or baseline risk, may significantly influence outcomes. Additionally, the protocols for NP‐guided therapy vary widely across studies, including differences in the frequency of NP measurements, target NP levels, and subsequent therapeutic interventions. In our analysis, we compare NP‐guided care exclusively with usual care, whereas McLellan et al. [20] in their meta‐analysis compared NP‐guided care with a combination of usual care and multidisciplinary care. This difference in selection criteria may contribute to the variations in our findings.

The standard of care for HF has improved significantly with the introduction of therapies such as sodium–glucose cotransporter 2 inhibitors and angiotensin receptor‐neprilysin inhibitors, potentially reducing the incremental value of NP‐guided strategies. Additionally, the open‐label design of many trials may have led to overlap in management between NP‐guided and usual care groups, as therapeutic decisions are often influenced by multiple clinical factors beyond NP levels. This overlap may help explain the neutral findings observed in recent studies. Future trials should ensure clearer therapeutic contrasts between intervention and control groups to more accurately assess the impact of biomarker‐guided approaches.

These results should also be interpreted within the broader context of the ongoing discussion around biomarker‐ versus guideline‐directed management. As noted by Fonarow in a JAMA editorial following the GUIDE‐IT trial, NT‐proBNP‐guided therapy did not improve outcomes compared to usual care, likely due to both groups receiving similar intensities of guideline‐recommended therapy [22]. This underscores the central importance of implementing optimal medical treatment, irrespective of biomarker levels. Nevertheless, our findings suggest that the role of NP guidance in the acute setting may still warrant further exploration, particularly when used to actively guide treatment decisions.

## 5 Limitations

Our study has several limitations that warrant consideration. First, the heterogeneity observed across the included trials, particularly for composite outcomes and adverse events, complicates the interpretation of our findings. This variability may be due to differences in study populations, HF severity, baseline risk, and treatment protocols across the included RCTs. Additionally, variations in the frequency of NP measurements, target levels, and therapeutic responses in the NP‐guided groups may have influenced the outcomes. The inclusion of the STRONG‐HF trial significantly impacted the heterogeneity of several endpoints, suggesting that study‐specific factors might play a critical role in the overall analysis. Besides, the primary intervention focused on rapid optimization of HF medications rather than therapeutic adjustments explicitly guided by serial NT‐proBNP levels. NT‐proBNP was used for monitoring purposes, and its clinical trajectory was not central to decision‐making. This distinction limits the extent to which STRONG‐HF can be interpreted as evidence for NP‐guided therapy, and this has been acknowledged accordingly in our analysis.

Moreover, the follow‐up periods varied between studies, ranging from 4 to 24 months, which could affect the long‐term applicability of the results. Finally, while we attempted to mitigate bias through robust methodology, the presence of moderate risk in several studies, as assessed by the RoB 2 tool, indicates the possibility of bias influencing the results.

## 6 Conclusion

In this meta‐analysis of 9 RCTs including 3992 patients with decompensated HF, NP‐guided therapy showed no consistent benefit over usual care in reducing mortality, hospitalizations, or improving quality of life. A significant advantage was observed only in time to all‐cause mortality, suggesting time‐dependent analysis may offer additional insight into long‐term outcomes. However, this was not mirrored in other endpoints, and the overall benefit remains uncertain. Further well‐designed trials using standardized protocols and time‐to‐event analyses are needed to clarify the role of NP‐guided therapy in the era of modern HF treatment.

## Author Contributions

**Luciana Gioli‐Pereira:** conceptualization, methodology, data curation, original draft preparation. **Eric Shih Katsuyama, Wilson Falco**, and **Christian Ken Fukunaga:** data curation, writing, software. **Camila Campos Grisa Padovese**, **Rafael Hortencio Melo**, and **Edielle de Sant'Anna Melo:** visualization, methodology, validation, supervision. **Silvana E. Ribeiro Papp** and **Fernando Bacal:** validation, supervision, reviewing, and editing. The authors take responsibility for all aspects of reliability and freedom from bias of the data presented and their discussed interpretation.

## Ethics Statement

This study is a systematic review and meta‐analysis based on previously published data. Therefore, ethical approval and informed consent were not required.

## Conflicts of Interest

The authors declare no conflicts of interest.

## Supporting information

Supporting Material Clin Cardiol.

## Data Availability

All data used in this study were extracted from published articles, which are publicly available. No new raw data were generated. The data sets used and/or analyzed during the current study are available from the corresponding author upon reasonable request.
