# Supplementary material for: Natriuretic Peptide‐Guided Therapy in Acute Decompensated Heart Failure: An Updated Systematic Review and Meta‐Analysis
Source: Clin Cardiol. 2025 Jun 4;48(6):e70165. doi: 10.1002/clc.70165 (PMC12135092; doi:10.1002/clc.70165)
Supplement: Supplementary file 1 — Supporting Material Clin Cardiol. [file CLC-48-e70165-s001.docx]

**SUPPLEMENTARY MATERIAL**

**Natriuretic peptide-guided therapy in acute decompensated heart failure: An updated systematic review and meta-analysis**

Luciana Gioli-Pereira, MD, pHD; Eric Shih Katsuyama, MD; Christian Ken Fukunaga; Wilson Falco; Camila Campos Grisa Padovese, MD; Rafael Hortencio Melo, MD; Edielle de Sant´Anna Melo, MD, pHD; Silvana E Ribeiro Papp, MD; Fernando Bacal, MD, pHD

**Table of Contents**

[**Supplemental Methods 1. PRISMA 2020 Main Checklist 3**](#_heading=h.30j0zll)

[**Supplemental Methods 2. PRISMA Abstract Checklist 3**](#_heading=h.1fob9te)

[**Supplemental Methods 3. Details of the Search Strategy 4**](#_heading=h.3znysh7)

[**Supplemental Methods 4. Outcomes Definitions 5**](#_heading=h.2et92p0)

[**Supplemental Methods 5. Kaplan Meier Curve Data Extraction 6**](#_heading=h.93yb4uk59sqb)

[**Supplemental Methods 6. Addressing Heterogeneity 7**](#_heading=h.obqehmk0kf7a)

[**Supplemental Table 1. Eligibility Criteria per study 8**](#_heading=h.g8lcwne5ji8k)

[**Supplemental Table 2. Additional Included Study’s Baseline Characteristics 13**](#_heading=h.ze4zrhysuk3b)

[**Supplemental Table 3. Definitions of Composite Outcome 15**](#_heading=h.1t3h5sf)

[**Supplemental Table 4. Target and titration therapy for the BNP Guided Group 17**](#_heading=h.4d34og8)

[**Supplemental Figure 1. Forest Plot for Time-to-event for all-cause mortality 21**](#_heading=h.17dp8vu)

[**Supplemental Figure 2. Forest plot for time to event composite endpoint 22**](#_heading=h.3rdcrjn)

[**Supplemental Figure 3. Forest Plot Cardiovascular Death endpoint 23**](#_heading=h.26in1rg)

[**Supplemental Figure 4. Forest Plot for Heart Failure Hospitalization 24**](#_heading=h.l12v8bwmdqz3)

[**Supplemental Figure 5. Forest Plot for Adverse Events
Figure 5A. Hypotension 25**](#_heading=h.qeuz4oxbo0cw)

**Figure 5B. Renal Impairment ……………………………………………………………………………….25**

[**Supplemental Figure 6. Sensitivity Analysis of the Primary Efficacy Endpoint 26**](#_heading=h.l7tnbwvudp7n)

[**Supplemental Figure 7. Trial Sequential Analysis for the Primary Endpoint 27**](#_heading=h.lnxbz9)

[**Supplemental Figure 8. Risk of Bias 2(RoB-2) of All Included Studies 28**](#_heading=h.i86fbsppo1la)

[**Supplemental Figure 9. Funnel Plot for the Primary Endpoint 29**](#_heading=h.35nkun2)

[**Supplemental References 30**](#_heading=h.aqe5hygonrr0)

# Supplemental Methods 1. PRISMA 2020 Main Checklist

| **Topic** | **No.** | **Item** | **Location where item is reported** |
| --- | --- | --- | --- |
| **TITLE** |  |  |  |
| **Title** | 1 | Identify the report as a systematic review. | Pg. 1; MS |
| **ABSTRACT** |  |  |  |
| **Abstract** | 2 | See the PRISMA 2020 for Abstracts checklist | Pg. 2; MS |
| **INTRODUCTION** |  |  |  |
| **Rationale** | 3 | Describe the rationale for the review in the context of existing knowledge. | Pg. 4; MS |
| **Objectives** | 4 | Provide an explicit statement of the objective(s) or question(s) the review addresses. | Pg. 4; MS |
| **METHODS** |  |  |  |
| **Eligibility criteria** | 5 | Specify the inclusion and exclusion criteria for the review and how studies were grouped for the syntheses. | Pg. 5; MS |
| **Information sources** | 6 | Specify all databases, registers, websites, organizations, reference lists and other sources searched or consulted to identify studies. Specify the date when each source was last searched or consulted. | Pg. 5; MS |
| **Search strategy** | 7 | Present the full search strategies for all databases, registers, and websites, including any filters and limits used. | Pg. 5; MS |
| **Selection process** | 8 | Specify the methods used to decide whether a study met the inclusion criteria of the review, including how many reviewers screened each record and each report retrieved, whether they worked independently, and if applicable, details of automation tools used in the process. | Pg. 5-6; MS |
| **Data collection process** | 9 | Specify the methods used to collect data from reports, including how many reviewers collected data from each report, whether they worked independently, any processes for obtaining or confirming data from study investigators, and if applicable, details of automation tools used in the process. | Pg. 6; MS |
| **Data items** | 10a | List and define all outcomes for which data were sought. Specify whether all results that were compatible with each outcome domain in each study were sought (e.g., for all measures, time points, analyses), and if not, the methods used to decide which results to collect. | Pg. 6; MS |
|  | 10b | List and define all other variables for which data were sought (e.g., participant and intervention characteristics, funding sources). Describe any assumptions made about any missing or unclear information. | Pg. 6; MS |
| **Study risk of bias assessment** | 11 | Specify the methods used to assess risk of bias in the included studies, including details of the tool(s) used, how many reviewers assessed each study and whether they worked independently, and if applicable, details of automation tools used in the process. | Pg. 7; MS |
| **Effect measures** | 12 | Specify for each outcome the effect measure(s) (e.g., risk ratio, mean difference) used in the synthesis or presentation of results. | Pg 6; MS |
| **Synthesis methods** | 13a | Describe the processes used to decide which studies were eligible for each synthesis (e.g., tabulating the study intervention characteristics and comparing against the planned groups for each synthesis (item 5)). | Table 1 |
|  | 13b | Describe any methods required to prepare the data for presentation or synthesis, such as handling of missing summary statistics, or data conversions. | Pg. 7 M.S. |
|  | 13c | Describe any methods used to tabulate or visually display results of individual studies and syntheses. | Pg. 7 M.S. |
|  | 13d | Describe any methods used to synthesize results and provide a rationale for the choice(s). If meta-analysis was performed, describe the model(s), method(s) to identify the presence and extent of statistical heterogeneity, and software package(s) used. | Pg. 7 M.S. |
|  | 13e | Describe any methods used to explore possible causes of heterogeneity among study results (e.g., subgroup analysis, meta-regression). | Pg. 7 M.S. |
|  | 13f | Describe any sensitivity analyses conducted to assess robustness of the synthesized results. | Pg. 7 M.S. |
| **Reporting bias assessment** | 14 | Describe any methods used to assess risk of bias due to missing results in a synthesis (arising from reporting biases). | Pg. 7 M.S. |
| **Certainty assessment** | 15 | Describe any methods used to assess certainty (or confidence) in the body of evidence for an outcome. | NA |
| **RESULTS** |  |  |  |
| **Study selection** | 16a | Describe the results of the search and selection process, from the number of records identified in the search to the number of studies included in the review, ideally using a flow diagram. | Figure 1 |
|  | 16b | Cite studies that might appear to meet the inclusion criteria, but which were excluded, and explain why they were excluded. | NA |
| **Study characteristics** | 17 | Cite each included study and present its characteristics. | Table 1 |
| **Risk of bias in studies** | 18 | Present assessments of risk of bias for each included study. | Fig. 9; Supplementary |
| **Results of individual studies** | 19 | For all outcomes, present, for each study: (a) summary statistics for each group (where appropriate) and (b) an effect estimate and its precision (e.g., confidence/credible interval), ideally using structured tables or plots. | Fig. 8-9; MS |
| **Results of syntheses** | 20a | For each synthesis, briefly summarize the characteristics and risk of bias among contributing studies. | Pg. 11; MS |
|  | 20b | Present results of all statistical syntheses conducted. If meta-analysis was done, present for each the summary estimate and its precision (e.g., confidence/credible interval) and measures of statistical heterogeneity. If comparing groups, describe the direction of the effect. | Pg. 8-11; MS |
|  | 20c | Present results of all investigations of possible causes of heterogeneity among study results. | Pg. 10; MS |
|  | 20d | Present results of all sensitivity analyses conducted to assess the robustness of the synthesized results. | Pg. 10; MS |
| **Reporting biases** | 21 | Present assessments of risk of bias due to missing results (arising from reporting biases) for each synthesis assessed. | Pg. 12; MS |
| **Certainty of evidence** | 22 | Present assessments of certainty (or confidence) in the body of evidence for each outcome assessed. | NA |
| **DISCUSSION** |  |  |  |
| **Discussion** | 23a | Provide a general interpretation of the results in the context of other evidence. | Pg. 11-14; MS |
|  | 23b | Discuss any limitations of the evidence included in the review. | Pg. 14; MS |
|  | 23c | Discuss any limitations of the review processes used. | Pg. 14; MS |
|  | 23d | Discuss implications of the results for practice, policy, and future research. | Pg. 14; MS |
| **OTHER INFORMATION** |  |  |  |
| **Registration and protocol** | 24a | Provide registration information for the review, including register name and registration number, or state that the review was not registered. | CRD42024579101 |
|  | 24b | Indicate where the review protocol can be accessed, or state that a protocol was not prepared. | <https://www.crd.york.ac.uk/PROSPERO/display_record.php?RecordID=579101> |
|  | 24c | Describe and explain any amendments to information provided at registration or in the protocol. | NA |
| **Support** | 25 | Describe sources of financial or non-financial support for the review, and the role of the funders or sponsors in the review. | None |
| **Competing interests** | 26 | Declare any competing interests of review authors. | None |
| **Availability of data, code and other materials** | 27 | Report which of the following are publicly available and where they can be found template data collection forms; data extracted from included studies; data used for all analyses; analytic code; any other materials used in the review. | NA |

^Abbreviations: MS, manuscript; sup., supplement.^

# Supplemental Methods 2. PRISMA Abstract Checklist

| **Topic** | **No.** | **Item** | **Reported?** |
| --- | --- | --- | --- |
| **TITLE** |  |  |  |
| **Title** | 1 | Identify the report as a systematic review. | Yes |
| **BACKGROUND** |  |  |  |
| **Objectives** | 2 | Provide an explicit statement of the main objective(s) or question(s) the review addresses. | Yes |
| **METHODS** |  |  |  |
| **Eligibility criteria** | 3 | Specify the inclusion and exclusion criteria for the review. | Yes |
| **Information sources** | 4 | Specify the information sources (e.g., databases, registers) used to identify studies and the date when each was last searched. | Yes |
| **Risk of bias** | 5 | Specify the methods used to assess risk of bias in the included studies. | No |
| **Synthesis of results** | 6 | Specify the methods used to present and synthesize results. | Yes |
| **RESULTS** |  |  |  |
| **Included studies** | 7 | Give the total number of included studies and participants and summarize relevant characteristics of studies. | Yes |
| **Synthesis of results** | 8 | Present results for main outcomes, preferably indicating the number of included studies and participants for each. If meta-analysis was done, report the summary estimate and confidence/credible interval. If comparing groups, indicate the direction of the effect (i.e., which group is favored). | Yes |
| **DISCUSSION** |  |  |  |
| **Limitations of evidence** | 9 | Provide a brief summary of the limitations of the evidence included in the review (e.g., study risk of bias, inconsistency and imprecision). | No |
| **Interpretation** | 10 | Provide a general interpretation of the results and important implications. | No |
| **OTHER** |  |  |  |
| **Funding** | 11 | Specify the primary source of funding for the review. | No |
| **Registration** | 12 | Provide the register name and registration number. | Yes |

# Supplemental Methods 3. Details of the Search Strategy

| **Search Strategy for each database** | |
| --- | --- |
| **Pubmed** | (“acute heart failure” OR “hospital setting” OR “heart failure” OR “heart failure with reduced ejection fraction” OR “heart failure with preserved ejection fraction” OR HFrEF OR HFpEF OR “acutely decompensated HF” OR “HF management” OR “acute setting” OR "Heart Failure"[Mesh] OR "Heart Failure, Diastolic"[Mesh] OR "Heart Failure, Systolic"[Mesh]) AND (“high intensity care” OR “care strategy” OR “NP-guided treatment” OR “NT-proBNP” OR BNP OR “B-type natriuretic peptide” OR “Natriuretic Peptide-guided treatment” OR biomarkers OR “cardiac biomarkers” OR “guided therapy” OR “biomarker-guided therapy”) AND (“clinical assessment” OR “usual care” OR “conventional therapy” OR “conventional treatment” OR “symptom-guided treatment”) AND (randomized controlled trial[pt] OR controlled clinical trial[pt] OR clinical trials as topic[mesh:noexp] OR trial[ti] OR random*[tiab] OR placebo*[tiab]) |
| **EMBASE** | (‘acute heart failure’ OR ‘hospital setting’ OR ‘heart failure’ OR ‘heart failure with reduced ejection fraction’ OR ‘heart failure with preserved ejection fraction’ OR HFrEF OR HFpEF OR ‘acutely decompensated HF’ OR ‘HF management’ OR ‘acute setting’ OR ‘Heart Failure’ OR ‘Heart Failure’) AND (‘high intensity care’ OR ‘care strategy’ OR ‘NP-guided treatment’ OR ‘NT-proBNP’ OR BNP OR ‘B-type natriuretic peptide’ OR ‘Natriuretic Peptide-guided treatment’ OR biomarkers OR ‘cardiac biomarkers’ OR ‘guided therapy’ OR ‘biomarker-guided therapy’) AND (‘clinical assessment’ OR ‘usual care’ OR ‘conventional therapy’ OR ‘conventional treatment’ OR ‘symptom-guided treatment’) (‘acute heart failure’ OR ‘hospital setting’ OR ‘heart failure’ OR ‘heart failure with reduced ejection fraction’ OR ‘heart failure with preserved ejection fraction’ OR HFrEF OR HFpEF OR ‘acutely decompensated HF’ OR ‘HF management’ OR ‘acute setting’ OR ‘Heart Failure’ OR ‘Heart Failure’) AND (‘high intensity care’ OR ‘care strategy’ OR ‘NP-guided treatment’ OR ‘NT-proBNP’ OR BNP OR ‘B-type natriuretic peptide’ OR ‘Natriuretic Peptide-guided treatment’ OR biomarkers OR ‘cardiac biomarkers’ OR ‘guided therapy’ OR ‘biomarker-guided therapy’) AND (‘clinical assessment’ OR ‘usual care’ OR ‘conventional therapy’ OR ‘conventional treatment’ OR ‘symptom-guided treatment’) AND ('controlled clinical trial'/exp OR (random* OR placebo*):ti,ab OR trial:ti) AND [embase]/lim |
| **Cochrane Library** | (“acute heart failure” OR “hospital setting” OR “acutely decompensated HF” OR “HF management” OR “acute setting”) AND (“high intensity care” OR “care strategy” OR “NP-guided treatment” OR “NT-proBNP” OR BNP OR “B-type natriuretic peptide” OR “Natriuretic Peptide-guided treatment” OR biomarkers OR “cardiac biomarkers” OR “guided therapy” OR “biomarker-guided therapy”) AND (“clinical assessment” OR “usual care” OR “conventional therapy” OR “conventional treatment” OR “symptom-guided treatment”) |

# Supplemental Methods 4. Outcomes Definitions

| **Outcomes and terms** | **Definition** |
| --- | --- |
| **All-Cause Mortality** | Death by any cause (1). |
| **Composite Outcome** | HF hospitalization and all-cause mortality |
| **Heart Failure**  **Hospitalization** | Hospital admission is due to any heart failure-related symptoms, lab/imaging findings, or physical signs. An investigator must report the hospitalization. Heart failure hospitalizations were also considered as a worsening in heart failure episodes (2). |
| **Cardiovascular Death** | CV deaths include those resulting from a myocardial infarction (MI), sudden cardiac death, heart failure (HF), stroke, cardiovascular procedures, cardiovascular bleeding, and other CV causes (3). |
| **Quality of Life** | Patient's overall well-being, encompassing physical, mental, and social aspects of health, particularly in the context of chronic cardiovascular conditions (4). |
| **Adverse Events** | Any unfavorable or unintended sign, symptom, or disease associated with the use of a drug or intervention, regardless of its causality. |

# Supplemental Methods 5. Kaplan Meier Curve Data Extraction

To collect the individual participant data (IPD), we followed Guyot et al. (5) method of reconstructing the IPD through the Kaplan-Meier Curve reports in some of the included studies. After downloading and digitizing the images of the Kaplan-Meier curves, the step function values and step timing were obtained, and the time-to-event data was calibrated using the total number of patients and number-at-risk tables. Finally, the individual patient survival data was obtained using the inverted Kaplan-Meier product limit equations, and the survival data was calculated using a Cox regression model.

# Supplemental Methods 6. Addressing Heterogeneity

To guarantee the robustness and certainty of our findings, we conducted a sensitivity analysis to identify potential outliers and influential trials for the primary endpoints. We used two methods to address heterogeneity: (1) we performed a leave-one-out analysis, which removes one study at a time to ensure that our results were not reliant on a single trial by also giving the residual heterogeneity after exclusion; and (2) a Baujat plot that is defined as a graphical method for detecting the sources of heterogeneity and evaluating the contribution of these sources to the overall result.

# Supplemental Table 1. Eligibility Criteria per study

#

| **Outcome** | **Eligibility Criteria** |
| --- | --- |
| **STRONG HF 2023**  **STRONG HF 2023**  ***(continued)*** | **"**INCLUSION CRITERIA: In order to be eligible to participate in this study, an individual must meet all of the following criteria:(1). Hospital admission within the 72 hours prior to screening for acute heart failure with dyspnea at rest and pulmonary congestion on chest X-ray, and other signs and/or symptoms of heart failure such as edema and/or positive rales on auscultation. (2) All measures within 24 hours prior to randomization of systolic blood pressure ≥ 100 mmHg, and of heart rate ≥ 60 bpm. (3) All measures within 24 hours prior to randomization of serum potassium ≤ 5·0 mEq/L(mmol/L).(4) Biomarker criteria for persistent congestion:(a) At Screening, NT-proBNP > 2,500 pg/mL.(b) At the time of Randomization (within 2 days prior to discharge), NT-proBNP > 1,500 pg/mL (to ensure the persistence of congestion) that has decreased by more than 10% compared to screening (to ensure the acuity of the index episode).(5) At 1 week prior to admission, at Screening, and at visit 2 (just prior to Randomization) either (a) ≤ ½ the optimal dose of ACEi/ARB/ARNi (see Table) prescribed, no beta-blocker prescribed, and ≤ ½ the optimal dose of MRA prescribed or (b) no ACEi/ARB/ARNi prescribed, ≤ ½ the optimal dose of beta-blocker prescribed, and ≤ ½ the optimal dose of MRA prescribed. (6) Written informed consent to participate in the study  EXCLUSION CRITERIA:An individual who meets any of the following criteria will be excluded from participation in this study:(1) Age < 18 or > 85 years; (2) Clearly documented intolerance to high doses of beta-blockers;(3) Clearly documented intolerance to high doses of RAS blockers (both ACEi and ARB);(4) Mechanical ventilation (not includingCPAP/BIPAP) in the 24 hours prior to screening.(5) Significant pulmonary disease contributing substantially to the patients’ dyspnea such as FEV1<1 liter or need for chronic systemic or nonsystemic steroid therapy, or any kind of primary right heart failure such as primary pulmonary hypertension or recurrent pulmonary embolism; (6) Myocardial infarction, unstable angina or cardiac surgery within 3 months, or cardiac resynchronization therapy (CRT) device implantation within 3 months, or percutaneous transluminal coronary intervention (PTCI), within 1 month prior to screening. (7) Index Event (admission for AHF) triggered primarily by a correctable etiology such as significant arrhythmia (e.g., sustained ventricular tachycardia, or atrial fibrillation/flutter with sustained ventricular response >130 beats per minute, or bradycardia with sustained ventricular arrhythmia <45 beats per minute), infection, severe anemia, acute coronary syndrome, pulmonary embolism, exacerbation of COPD, planned admission for device implantation or severe nonadherence leading to very significant fluid accumulation prior to admission and brisk diuresis after admission. Troponin elevations without other evidence of an acute coronary syndrome are not exclusion.(8) Uncorrected thyroid disease, active myocarditis, or known amyloid or hypertrophic obstructive cardiomyopathy.(9) History of heart transplant or on a transplant list,or using or planned to be implanted with a ventricular assist device. (10) Sustained ventricular arrhythmia with syncopal episodes within the 3 months prior to screening that is untreated.(11) Presence at Screening of any hemodynamically significant valvular stenosis or regurgitation, except mitral or tricuspid regurgitation secondary to left ventricular dilatation, or the presence of any hemodynamically significant obstructive lesion of the left ventricular outflow tract.(12.) Active infection at any time during the AHF hospitalization prior to Randomization based on abnormal temperature and elevated WBC or need for intravenous antibiotics.(13.) Stroke or TIA within the 3 months prior toScreening.(14.) Primary liver disease considered to be life threatening.(15) Renal disease or eGFR < 30 mL/min/1·73m2 (as estimated by the simplified MDRD formula) at Screening or history of dialysis.(16) Psychiatric or neurological disorder, cirrhosis, or active malignancy leading to a life expectancy < 6 months.(17) Prior (defined as less than 30 days from screening) or current enrollment in a CHF trial or participation in an investigational drug or device study within the 30 days prior to screening;(18) Discharge for the AHF hospitalization anticipated to be > 14 days from admission, or to a long-term care facility. Randomization must occur within 12 days following admission and within 2 days prior to anticipated discharge. (19) Inability to comply with all study requirements, due to major comorbidities, social or financial issues, or a history of noncompliance with medical regimens, that might compromise the patient’s ability to understand and/or comply with the protocol instructions or follow-up procedures.(20) Pregnant or nursing (lactating) women.  *Supplementary Text; Table 2* |
| **Berger et al. 2010** | "1) clinical signs and symptoms of cardiac decompensation during the present hospitalization; 2) New York Heart Association functional class III or IV at admission; and 3) cardiothoracic ratio 0.5 or left ventricular ejection fraction 40% as documented by echocardiography."  *Full Text. page 2* |
| **Guide IT 2017** | Patients were eligible for enrollment if they had chronic HFrEF with an ejection fraction of 40% or less, a history of a prior HF event (hospitalization for HF, emergency department visit for HF, or outpatient treatment with intravenous diuretics for HF) within the prior 12 months, and an NT-proBNP level of more than 2000 pg/mL or BNP of more than 400 pg/mL within the prior 30 days. Patients were excluded if they had an acute coronary syndrome or revascularization procedure within the prior 30 days, cardiac resynchronization therapy within the prior 3 months, end-stage renal disease, or anticipated heart transplant or mechanical cardiac support within the next 12 months. In accordance with National Institutes of Health policy, patient-reported race/ethnicity information was collected using fixed categories.  *Full Text; page. 2 Study Participants Section* |
| **Karlström et al. 2011** | “Patients older than 18 years with verified systolic HF and a left ventricular ejection fraction (LVEF) <40% (assessed within the last 6 months), New York Heart Association (NYHA) class II–IV, signs and/or symptoms of worsening HF within the last month (requiring hospitalization and/or intravenous diuretic treatment, metolazone, or increased daily doses of diuretics and/or need of intravenous inotropic support) were recruited. The patients were required to have elevated plasma concentrations of BNP (>150 ng/L for those aged <75 years, and >300 ng/L for those aged >75 years).  The patients were required to have ongoing standard HF treatment according to guidelines, defined as basic treatment with angiotensin-converting enzyme (ACE) inhibitors (ACEI) or angiotensin II receptor blockers (ARB), beta-blockers (BB), and diuretics, if fluid retention existed. In addition, they could also be treated with aldosterone antagonists (AA) and/or digoxin.”  *Full Text; Pag. 2* |
| **Lainchbury et al. 2009** | “Inclusion required age 18 years and symptomatic CHF, defined by Framingham criteria (15) and satisfying European Society of Cardiology diagnostic guidelines (16), precipitating admission, and ability to give informed consent. Immediate pre-randomization plasma NT-proBNP levels had to exceed 50 pmol/l (400 pg/ml). Exclusion criteria included active myocarditis/pericarditis, life expectancy due to noncardiovascular disease of 24 months, severe hepatic or pulmonary disease (forced expiratory volume in 1 s of 1 l), severe renal impairment (plasma creatinine 250 mol/l), severe valvular disease, or candidacy for cardiac transplantation. Recruitment deliberately included elderly patients and patients with a preserved LVEF.”  *Full Text; Pag. 2* |
| **PRIMA II 2017**  **PRIMA II 2017**  ***(continued)*** | **"Inclusion criteria:** (1)Admission for acute decompensated heart failure;(2)NT-proBNP levels of >1700 ng/L (>200 pmol/L) within 24 h of hospital admission  Exclusion criteria  (1)Severe chronic obstructive pulmonary disease with forced expiratory volume in 1 s of <1 L; (2) Pulmonary embolism within 1 mo before admission and pulmonary; (3)hypertension not caused by left ventricle dysfunction; (4)Patients undergoing continuous ambulant peritoneal dialysis/patients on hemodialysis; (5)Patients with planned coronary artery bypass grafting, percutaneous coronary intervention, cardiac resynchronization therapy, or valvular surgery before admission (until 1 day before admission);(6) Patients with planned coronary artery bypass grafting, percutaneous coronary intervention, cardiac resynchronization therapy, or valvular surgery during admission until the moment of randomization; (7)Patients in cardiogenic shock at admission requiring invasive treatment; (8)Patients with a history of ST-segment elevated myocardial infarction, coronary artery bypass grafting, percutaneous coronary intervention, cardiac resynchronization therapy, or valvular surgery within 1 mo before admission; (9) Signed informed consent for any current interventional study; (10)Presence of severe non cardiac related life-threatening disease before inclusion with an expected survival of <6 mo after inclusion; (11)Unwillingness to give or mental or physical status not allowing written informed consent; and (12) Circumstances that prevent follow-up (no permanent home address,transient, etc)"  *Full Text; Page 2; Table 1* |
| **PRIMA 2010** | "To be included, patients had to be hospitalized for decompensated, symptomatic HF, fulfilling the European Society of Cardiology (ESC) diagnostic guideline criteria for acute HF (9). In addition, NT-proBNP levels at admission were required to be at least 1,700 pg/ml, as additional objective evidence of HF (1).  Exclusion criteria were: life-threatening cardiac arrhythmias during the index hospitalization, urgent invasive or surgical intervention performed or planned during the index hospital admission, severe chronic obstructive pulmonary disease with a forced expiratory volume in 1 s (FEV1) of 1 l/s, pulmonary embolism less than 3 months prior to admission,pulmonary hypertension not caused by left ventricular systolic dysfunction, a non–HF-related expected survival of less than 1 year, and patients undergoing hemodialysis or continuous ambulant peritoneal dialysis. A lesser degree of renal dysfunction was not an exclusion criterion."  *Full Text; Page 3 Study design and study population.* |
| **TIME CHF** | The study was conducted at 15 centers in Switzerland and Germany and included patients aged 60 years or older with dyspnea (New York Heart Association [NYHA] class ≥II with current therapy), a history of hospitalization for heart failure within the last year, and an N-terminal BNP level of 400 pg/mL or higher (to convert to ng/L, multiply by 1.0) in patients younger than 75 years and a level of 800 pg/mL or higher in patients aged 75 years or older.  *Full Text; Page 2* |
| **Troughton et al. 2000** | "Patients aged 35–85 were recruited after hospital admission with decompensated heart failure or from a specialist cardiology outpatient clinic. All had impaired left-ventricular systolic function (left-ventricular ejection fraction <40% on 2- dimensional echocardiography), established symptomatic heart failure (New York Heart Association [NYHA] class II–IV), and were treated with ACE inhibitors, loop diuretic with or without digoxin"  *Full Text; Page 2* |

# Supplemental Table 2. Additional Included Study’s Baseline Characteristics

| **Study** | **Country** | **LVEF < 50%**  **N (%)** | **Blinding** | **Usual Care Therapy** | | | | |
| --- | --- | --- | --- | --- | --- | --- | --- | --- |
|  |  |  |  | **ARB** | **SGLT-2** | **BB** | **ACEi** | **Mineralocorticoid** |
| **STRONG HF 2023** | Argentina, Austria, Bulgaria, Columbia, France,  Hungary, Israel, Mozambique, Nigeria,  Russia, Serbia,  Slovakia, South Africa, and Tunisia | 912 (85) | Open-label | X | X | X | X | X |
| **Berger et al. 2010** | Austria | 278 (100) | Open-label | X |  | X | X | X |
| **Guide IT 2017** | USA  Canada | 894 (100) | Open-label | X |  | X | X | X |
| **Karlström et al. 2011** | Sweden and Norway | 279 (100) | Open-label | X |  | X | X | X |
| **Lainchbury et al. 2009** | New Zeland | NA | Double-Blind | X |  | X | X | X |
| **PRIMA II 2017** | Netherlands  Portugal  Spain | **^#^**243 (72) | Open Label | X |  | X | X | X |
| **PRIMA 2010** | Dutch | **^#^**229 (66) | Open Label | X |  | X | X | X |
| **TIME CHF 2019** | Germany and Switzerland | NA | Single-Blind | X |  | X | X | X |
| **Troughton et al. 2000** | New Zealand | 69 (100) | Double-blind |  |  |  | X | X |

*Abbreviations: ACEi: Angiotensin-Converting Enzyme Inhibitors; ARB-II: Angiotensin II receptor blockers; BB: Beta Blocker, LVEF: Left Ventricular Ejection Fraction; SGLT-2: sodium-glucose cotransporter-2 inhibitors; NA: NA= not available.*

***^#^*** *LVEF ≤ 45%*

# Supplemental Table 3. Definitions of Composite Outcome

| **Outcome** | **Definition** |
| --- | --- |
| **STRONG HF 2023** | "The primary endpoint of the trial (as amended on Jan 11, 2021) was 180-day heart failure readmission or all-cause death, considering only the first occurrence of these events per patient…"  *Full Text; Page 4; Outcomes Section* |
| **Berger et al. 2010** | "The analyzed end points included HF rehospitalization, duration of time it takes to reach the combined end point of death and HF rehospitalization, the first HF rehospitalization, and death."  *Full Text; Page 4; Outcomes Data Section* |
| **Guide IT 2017** | "The primary outcome was a composite of time-to-first HF hospitalization or death from cardiovascular causes."  *Full Text; Page 2; Study Outcome Section* |
| **Karlström et al. 2011** | “The primary outcome variable was a composite of death due to any cause, need for hospitalization and worsening HF. Worsening HF was defined as a need to increase diuretics orally or intravenously but no need for hospitalization. “  *Full Text; Page 2* |
| **Lainchbury et al. 2009** | “Primary outcomes included all-cause mortality and the composite of death plus hospitalization for heart failure.”  *Full Text; Page 3* |
| **PRIMA II 2017** | "The first primary endpoint was a composite end point of readmission for HF and all-cause mortality in 180 days after randomization…"  *Full Text; Page 3; Study Outcome Section* |
| **PRIMA 2010** | "…the combined end points of total and cardiovascular morbidity and mortality…"  *Full Text; Page 4; Definition of study endpoints section* |
| **Troughton et al. 2000** | “Heart failure or death”  *Full Text; Page 3; Figure 2* |

# Supplemental Table 4. Target and titration therapy for the BNP Guided Group

#

| **Outcome** | **Definition** |
| --- | --- |
| **STRONG HF**  **2023** | "For patients randomly assigned to the high-intensity care group, treatment followed an algorithm combining optimisation of oral heart failure therapies and frequent visits, including circulating NT-proBNP measures, to assess congestion. For patients in this group, the first dose adjustment occurred just after randomisation (within 2 days before anticipated hospital discharge), when patients were prescribed medical therapy with B blockers, renin-angiotensin blockers (ie, ACE inhibitors [or ARBs if intolerant to ACE inhibitors] or ARN inhibitors), and mineralocorticoid ~receptor antagonists adjusted to at least half the optimal doses. Doses considered to be optimal are provided in the appendix (p 5). Patients were assessed by the study team at 1,2, 3, and 6 weeks after randomisation (ie, baseline). Additionally, at 2 weeks after randomisation, up-titration to full optimal doses of B blockers; ACE inhibitors,ARBs, or ARN inhibitors; and the mineralocorticoid receptor antagonist should have been reached if safe."  *Full Text, page 3* |
| **Berger et al.**  **2010** | "In patients with a discharge NT-proBNP level 2,200pg/ml, ambulatory visits at a CHF specialist were performed at least every 2 weeks (except at scheduled nurse visits) in addition to MC to optimize medical therapy as quickly as possible. When NT-proBNP fell below 2,200 pg/ml 3 or 6 months after discharge, patients were followed similarly to those in the MC group. In patients with an ongoing elevated NT-proBNP 2,200 pg/ml, the every-2-weeks visits were continued until maximal recommended or tolerated doses of CHF therapy were established, following which the time interval between visits was increased to 3 months. The HF specialist used NT-proBNP in addition to other clinical and laboratory parameters for integrated clinical management (e.g., adaptation of diuretic regimen, rate of dose increase for neurohormonal antagonists, schedule of visits)…"  *Full Text; Page 3, Methods Section* |
| **Guide IT 2017** | "For patients randomized to the NT-proBNP-guided strategy, clinicians were instructed to titrate HF therapy to target an NT-proBNP level of less than 1000 pg/mL. Specific adjustments of therapy for individual patients were at the discretion of the treating physician, but sites were encouraged to prioritize titration of neurohormonal antagonists over diuretics unless there was clinical evidence of congestion or volume overload." "All patients in either group also had blinded NT-proBNP concentrations measured in a core laboratory at each study visit. For patients in either group, investigators were provided with the most recent American Heart Association (AHA)/American College of Cardiology (ACC) practice guidelines for the management of HF and specific information on target doses of proven medical therapies. After an initial visit at 2 and 6 weeks, visits occurred every 3 months throughout the remainder of the study. After therapy adjustment for HF (whether driven by NT-proBNP levels or clinical reasons), patients had a 2-week follow-up visit for reassessment.  *Full Text; Pag.2 Randomization and Treatment Assignments Section* |
| **Karlström et al. 2011** | “In the BNP-guided group, medical treatment was guided by the plasma concentration of BNP. The goal was to reduce BNP levels to <150 ng/L in patients aged <75 years and <300 ng/L in patients aged ≥75 years. Treatment recommendations in order to reduce elevated BNP levels or signs/symptoms of worsening HF were suggested according to the following schedule: increase ACEI/ARB to maximally tolerated or to target dose according to guidelines; increase BB to maximally tolerated or to target dose according to guidelines; add AA in low dose (spironolactone 25 mg); add ARB and increase to target dose according to guidelines; increase ACEI/ARB to up to twice the target dose; increase BB to up to twice the target dose; increase spironolactone to up to 50 mg. The adjustment of loop diuretic dose was left to the discretion of the investigator. The patients were made aware of their BNP value in order to increase motivation to adhere to treatment.”  *Full Text; Pag.2 Brain natriuretic peptide-guided group (BNP-group) on Methods Section* |
| **Lainchbury et al. 2009** | “For the NT-proBNP group, adjustments in medications and additional follow-up visits were triggered by an NT-proBNP level 150 pmol/l and/or a heart failure score 2.0 according to instructions by 1 investigator (J.G.L.) who did not undertake the clinical assessments. When results fell below both thresholds, treatment was not altered. Serial assessments were conducted for the CG group.”  *Full Text; Pag.2 and 3 Procedures Section* |
| **PRIMA II 2017** | Levels were revealed to the treating physicians for patients in the NT-proBNP-guided group, and it was possible to plan discharge and follow-up when NT-proBNP had decreased >30% already at the day of randomization. Patients in the NT-proBNP group in whom NT-proBNP had not decreased ≥30% from the admission value entered a predefined algorithm consisting of several steps, including additional NT-proBNP measurements and suggested interventions to try to attain the desired >30% reduction in NT-proBNP levels. Briefly, suggested interventions were titration of HF medication (angiotensin converting enzyme [ACE]-inhibitors, β-blockers, and mineralocorticoid receptor antagonists [MRAs]) in eligible patients,20 invasive therapies such as implantation of cardiac resynchronization therapy if indicated,20 diagnostic coronary angiography when there was a suspicion of coronary ischemia, and electric cardioversion for patients with new-onset atrial fibrillation (see separately published design paper for details on the algorithm19). Further therapy adjustments were left at the discretion of the treating physician (ie, diuretics when it was felt that the patient was still or again became congested). When patients were randomized to NT-proBNP-guided therapy and had a reduction of <30% in NT-proBNP at randomization, the study coordinator contacted the site for therapy advice. In addition, reasons for not initiating or titrating HF medication had to be reported by the treating physician.  *Full Text; Page 3; Study Patients, Group Assignments, and Interventions* |
| **PRIMA 2010** | Treatment in the NT-proBNP–guided group was guided by the combination of clinical assessment and NT-proBNP levels. The individual NT-proBNP target value was set at the lowest level at discharge or at 2 weeks follow-up. If at subsequent outpatient visits, NT-proBNP levels were more than 10% with a minimum of 850 pg/ml above this individual target level, NT-proBNP level was considered “off-target,” and therapy was intensified according to the ESC HF treatment guidelines (10). In this treatment group, an electronic case record form indicated at each visit whether NT-proBNP levels were off-target and indicated whether intensification was necessary.  *Full Text; page 3; Study design and study population section* |
| **TIME CHF** | Medical therapy was prescribed according to current European Society of Cardiology and American College of Cardiology/American Heart Association guidelines with predefined escalation rules simulating clinical practice to reduce either symptoms to dyspnea NYHA class of II or less (in the symptom-guided group) or N-terminal BNP levels to less than 2 times the upper limit of normal less than 400 pg/mL in patients younger than 75 years and less than 800 pg/mL in patients aged 75 years or older-and NYHA class of II or less (in the N-terminal BNP-guided group). |
| **Troughton et al. 2000** | "The treatment target in the clinical group was clinically compensated heart failure according to an objective score (heart-failure score <2), and in the BNP group, N-BNP below 200 pmol/L (which corresponds to the concentration of BNP-32 that discriminated decompensated from compensated heart failure in an earlier study). If these targets were not achieved, drug treatment was intensified according to a strict and predetermined stepwise protocol comprising: maximisation of ACE inhibitors (up to enalapril equivalent of 20 mg twice a day); increase in loop diuretic to furosemide 500 mg twice a day; addition of digoxin up to 0·25 mg/day; additional diuretic (spironolactone 25–50 mg once a day, then metolazone 2·5–5 mg once a day); then additional vasodilator (isosorbide mononitrate 60–120 mg once a day then felodipine 2·5–5 mg once a day). Patients in either group not meeting treatment targets were reassessed at 2-week intervals (by an investigator unaware of allocation) and treatment intensified (by the investigator who did know the allocation) until targets were met, at which point 3-month reviews were resumed."  *Full Text; Page 2. Methods Section* |

# Supplemental Figure 1. Forest Plot for Time-to-event for all-cause mortality

**
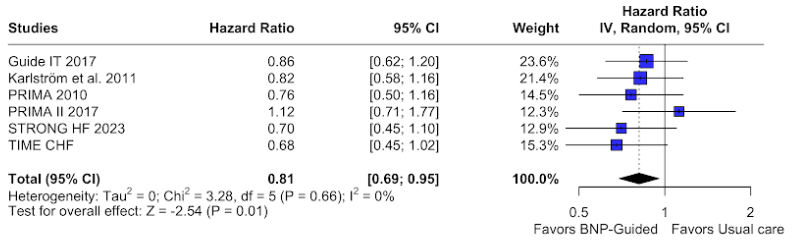
**

*Legend: In patients with acute decompensated heart failure, BNP-Guided therapy was significantly associated with a change in time to event all-cause mortality endpoint compared to usual care. Abbreviations: BNP-Brain Natriuretic Peptide; CI: Confidence Interval; IV: Inverse-Variance*

# Supplemental Figure 2. Forest plot for time to event composite endpoint

**
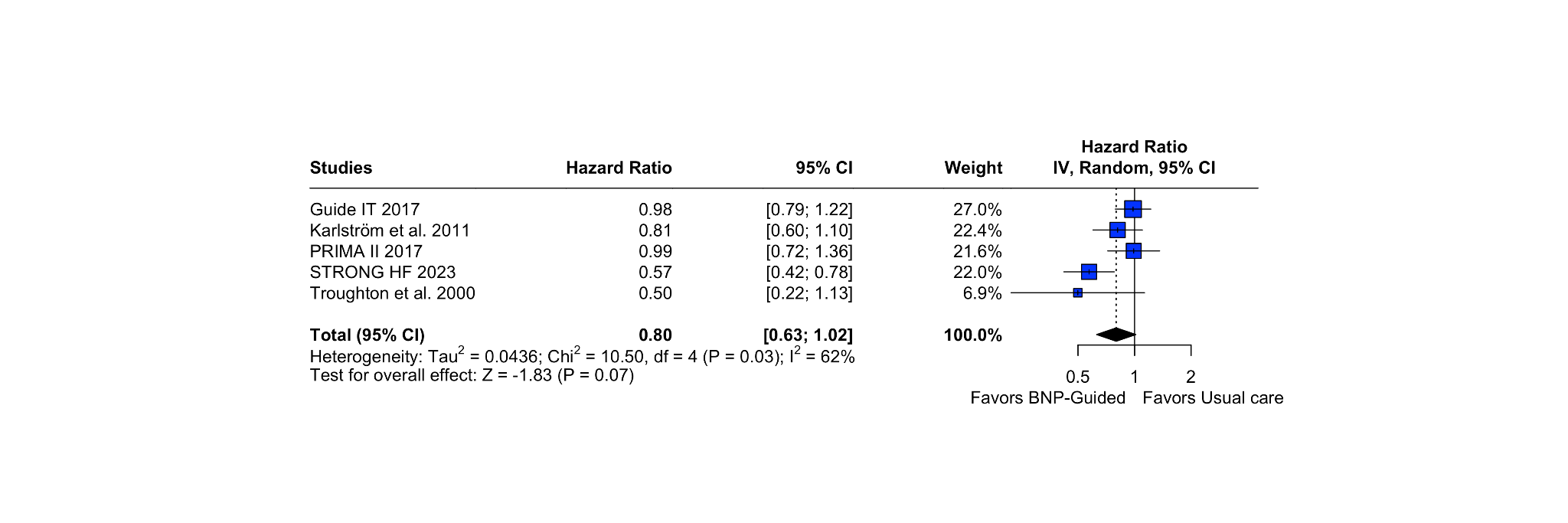
**

*Legend: In patients with acute decompensated heart failure, BNP-Guided therapy was not significantly associated with a change in risk of composite endpoint compared to usual care. Abbreviations: BNP-Brain Natriuretic Peptide; CI: Confidence Interval; MH:Mantel-Haenszel; RR: Risk Ratio*

# Supplemental Figure 3. Forest Plot Cardiovascular Death endpoint

**
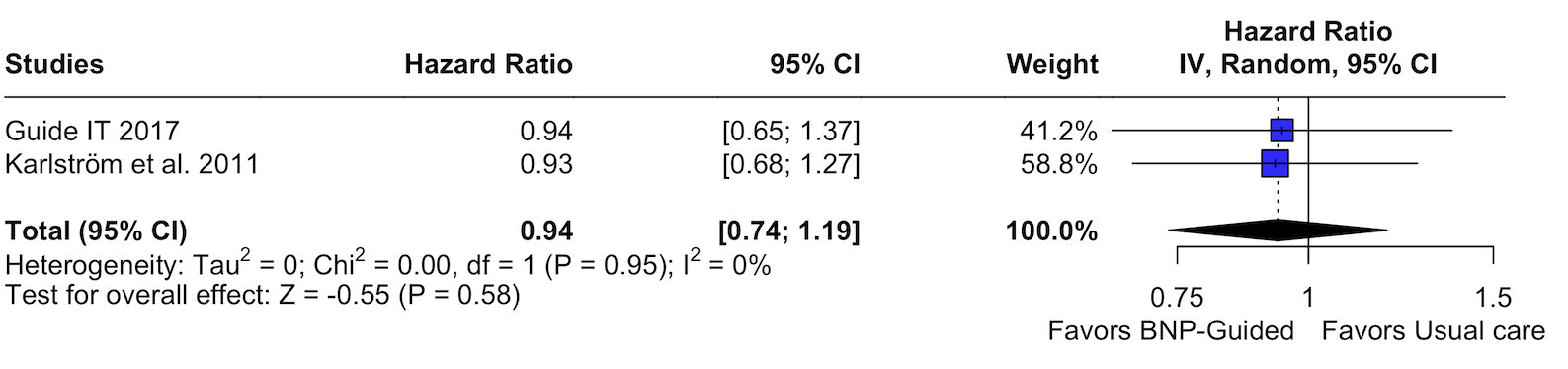
**

*Legend: In patients with acute decompensated heart failure, BNP-Guided therapy was not significantly associated with cardiovascular death endpoint compared to usual care. Abbreviations: BNP-Brain Natriuretic Peptide; CI: Confidence Interval; IV: Inverse-Variance*

# Supplemental Figure 4. Forest Plot for Heart Failure Hospitalization

**
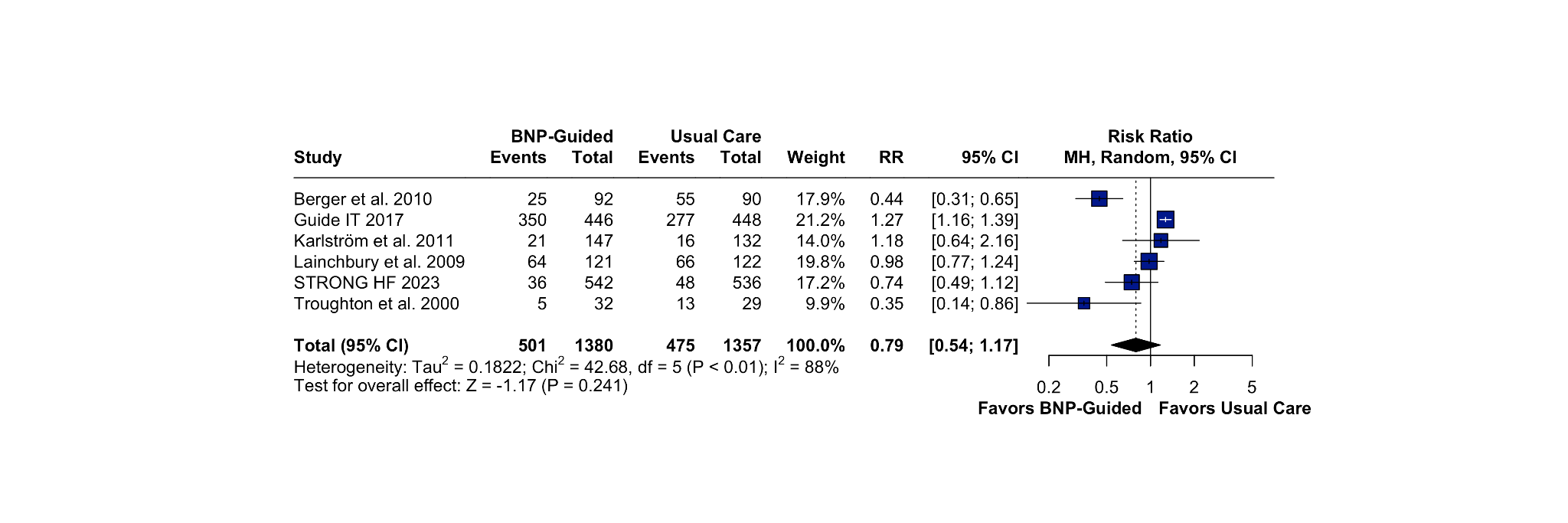
**

*Legend: In patients with acute decompensated heart failure, BNP-Guided therapy was not significantly associated with HF Hospitalization endpoint compared to usual care. Abbreviations: BNP-Brain Natriuretic Peptide; CI: Confidence Interval; MH:Mantel-Haenszel*

# Supplemental Figure 5. Forest Plot for Adverse Events Figure 5A. Hypotension

**
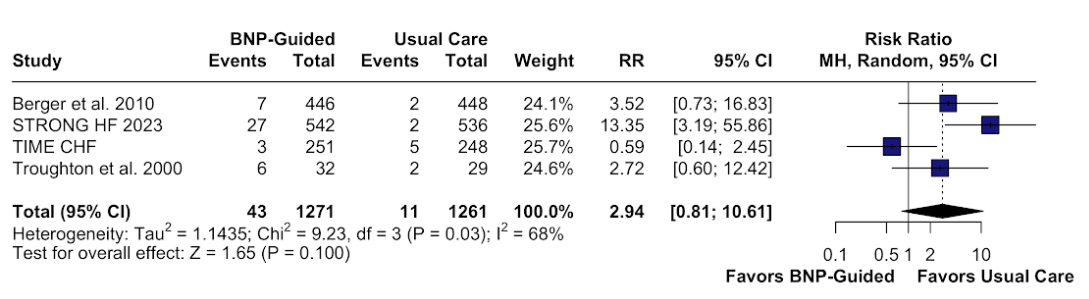
**

*Legend: There was no significant change with the hypotension endpoint compared to the usual care in patients with decompensated heart failure. Abbreviations: BNP-Brain Natriuretic Peptide; CI: Confidence Interval; MH:Mantel-Haenszel*

**Figure 5B.** Renal Impairment

**
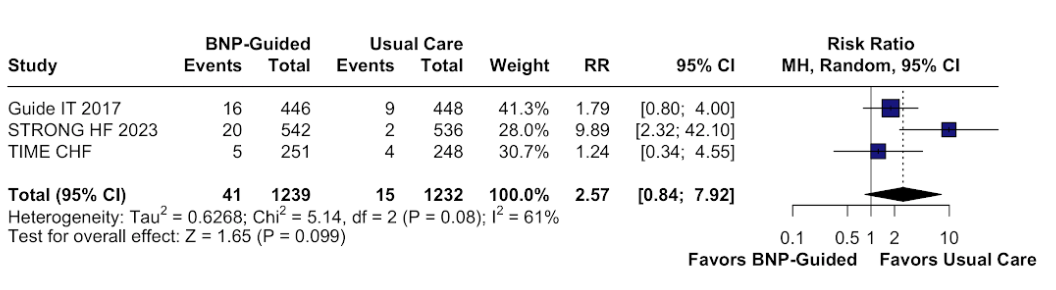
**

*Legend: There was no significant change with the renal impairment endpoint compared to usual cae in patients with decompensated heart failure. Abbreviations: BNP-Brain Natriuretic Peptide; CI: Confidence Interval; MH:Mantel-Haenszel*

# Supplemental Figure 6. Sensitivity Analysis of the Primary Efficacy Endpoint

**Figure 6A.** Leave-one-out method for the primary endpoint

**
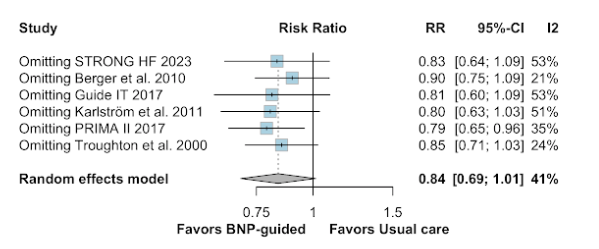
**

*Legend: Abbreviations: BNP-Brain Natriuretic Peptide; CI: Confidence Interval; RR:Risk Ratio*

**Figure 6B.** Baujat method for the primary endpoint

**
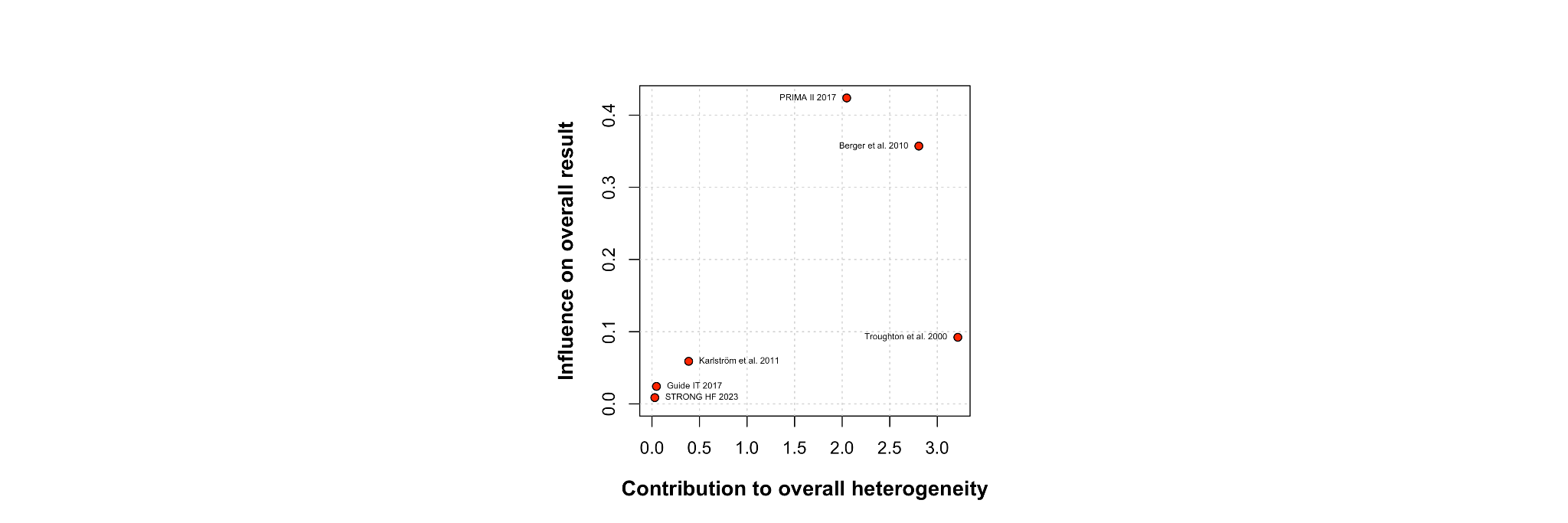
**

*Legend: Baujat Plot suggests PRIMA II 2017, Berg et al.2010, and Troughton et al. 2000 are possible outliers for the all-cause mortality endpoint.*

# Supplemental Figure 7. Trial Sequential Analysis for the Primary Endpoint


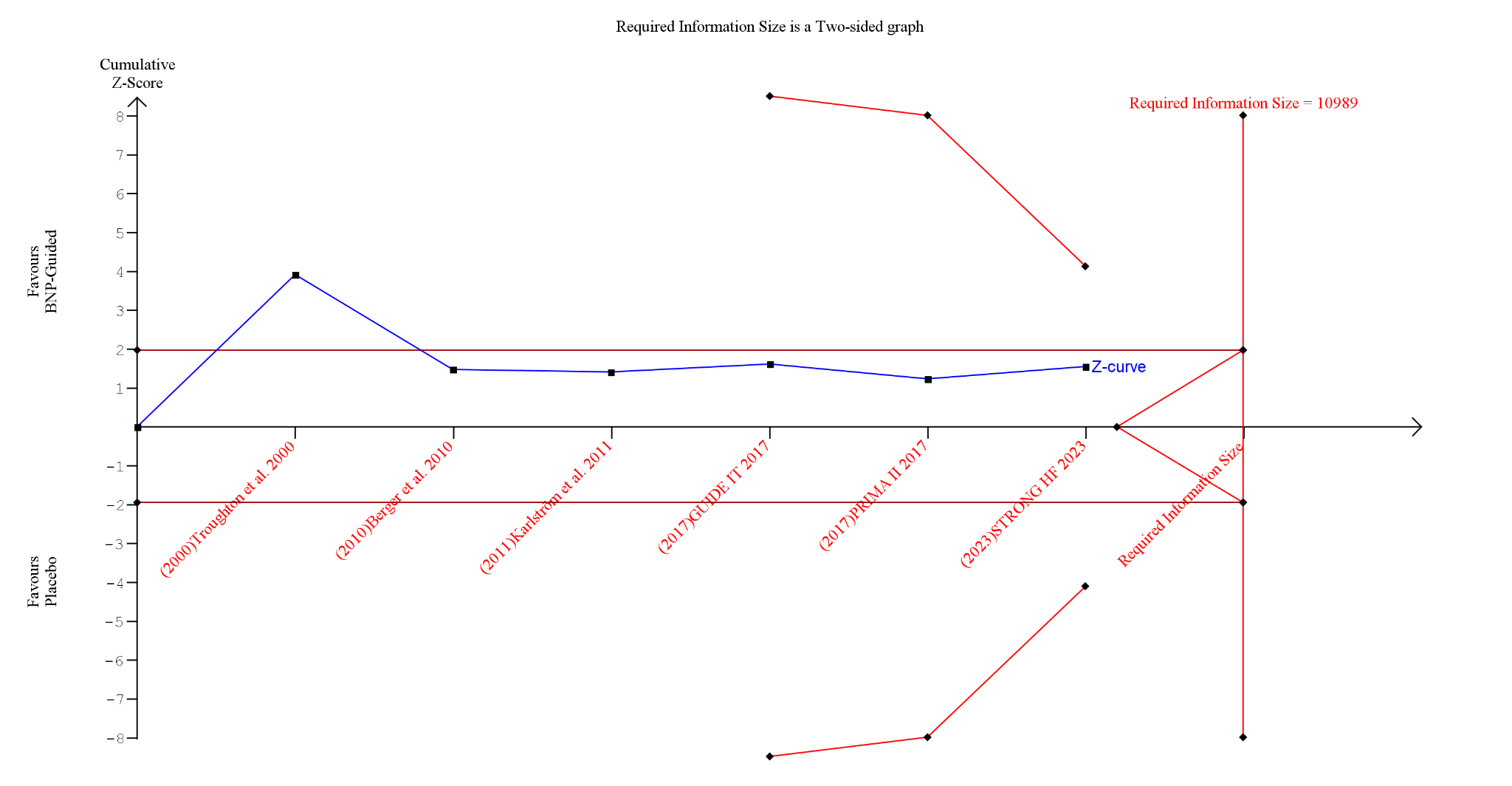


# Supplemental Figure 8. Risk of Bias 2(RoB-2) of All Included Studies

**Figure 8A.** “Traffic light” plot of the domain-level judgments for each study


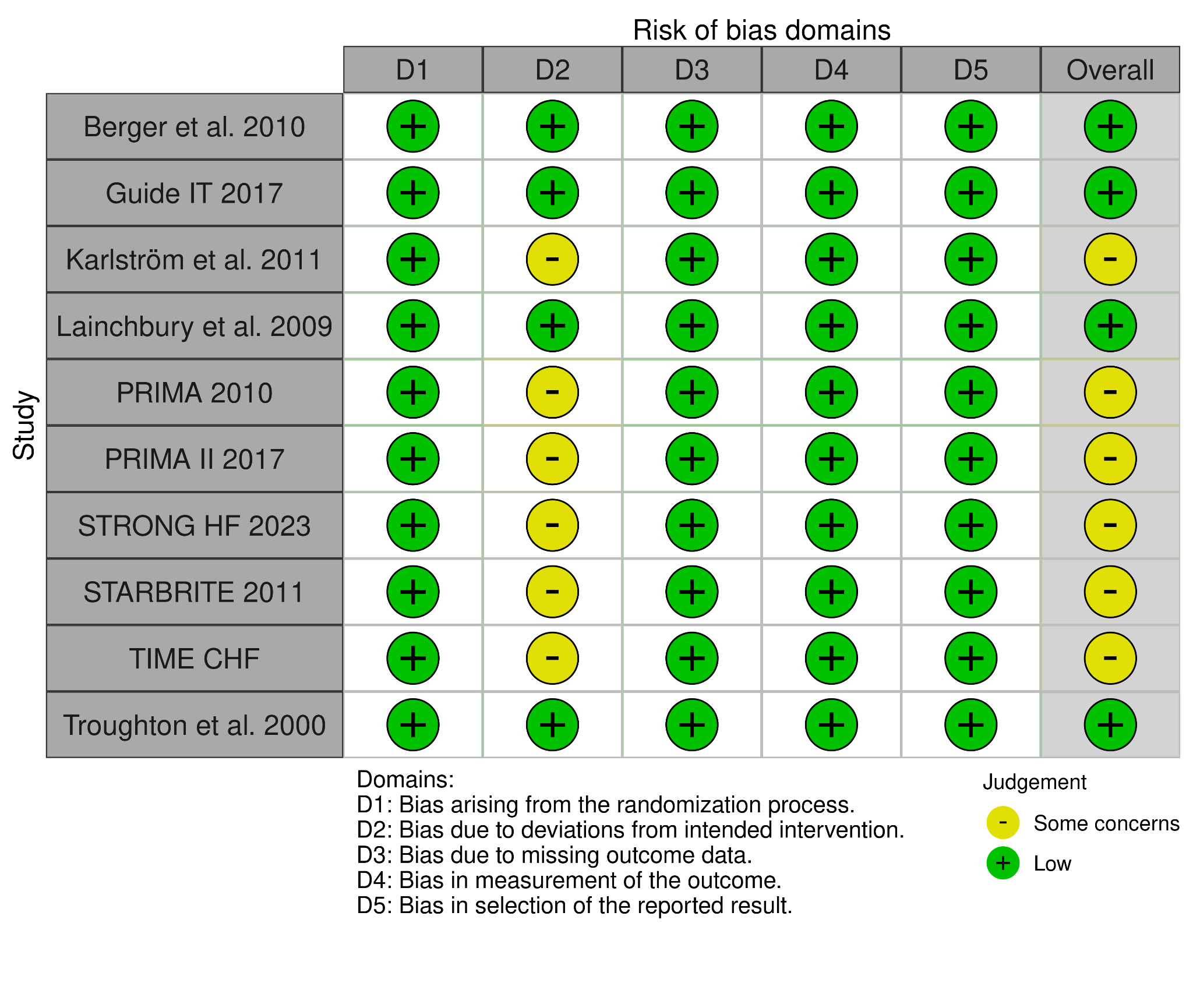


**Figure 8B.** Summary of overall weighted bar plot of risk-of-bias judgments within each bias domain.


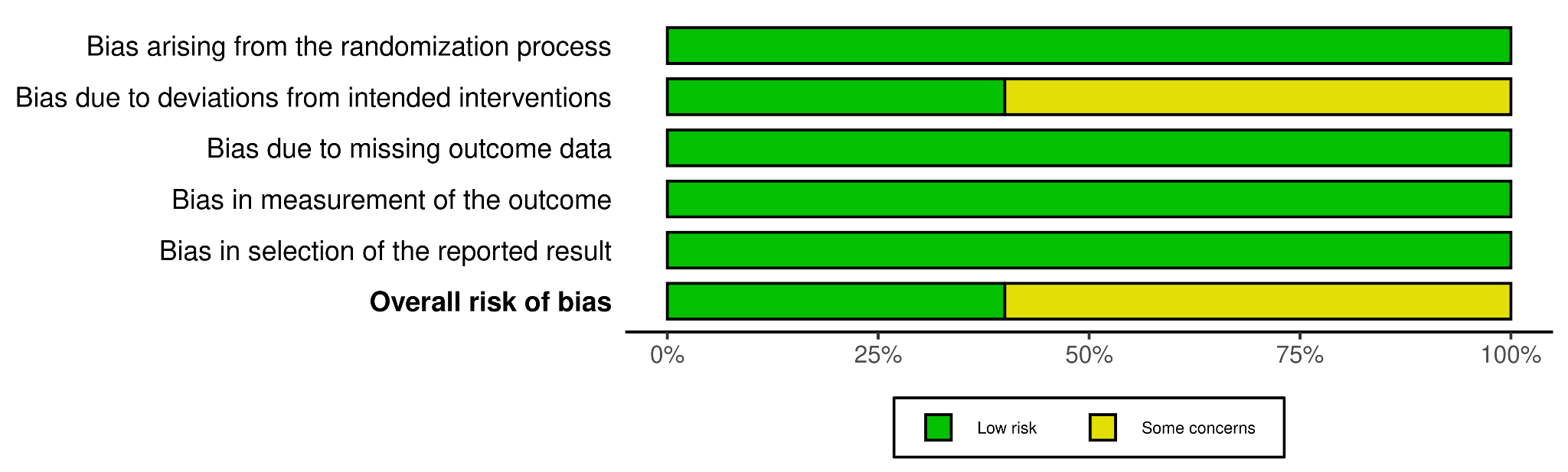


# Supplemental Figure 9. Funnel Plot for the Primary Endpoint
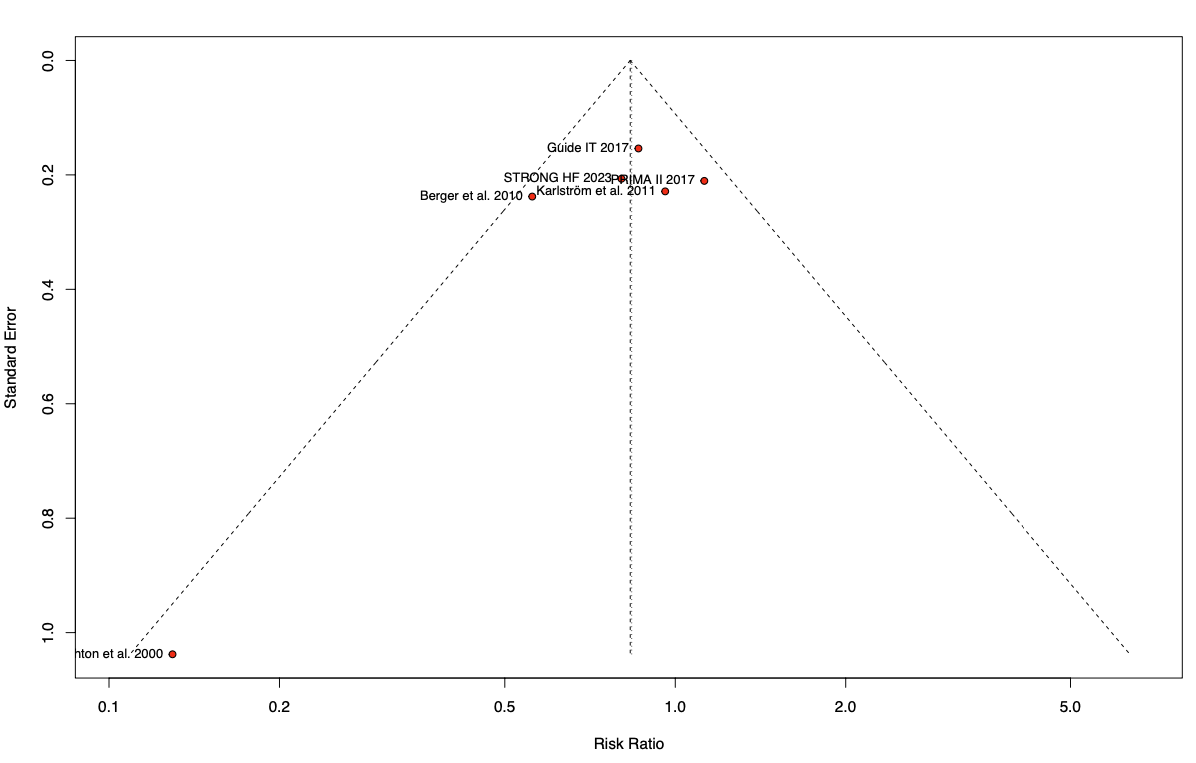


*Legend: Funnel plot shows a symmetrical distribution of similar-weight studies suggesting no small study effect.*

# Supplemental References

1. The Health Consequences of Smoking—50 Years of Progress: A Report of the Surgeon General. [Internet]. Centers for Disease Control and Prevention (US; 2014. Available from: https://www.ncbi.nlm.nih.gov/books/NBK294300/

2. Agarwal MA, Fonarow GC, Ziaeian B. National Trends in Heart Failure Hospitalizations and Readmissions From 2010 to 2017. JAMA Cardiol. 2021 Aug 1;6(8):952.

3. Hicks KA, Mahaffey KW, Mehran R, Nissen SE, Wiviott SD, Dunn B, et al. 2017 Cardiovascular and Stroke Endpoint Definitions for Clinical Trials. J Am Coll Cardiol. 2018 Mar;71(9):1021–34.

4. Rabin R, Charro FD. EQ-SD: a measure of health status from the EuroQol Group. Ann Med. 2001 Jan;33(5):337–43.

5. Guyot P, Ades A, Ouwens MJ, Welton NJ. Enhanced secondary analysis of survival data: reconstructing the data from published Kaplan-Meier survival curves. BMC Med Res Methodol. 2012 Dec;12(1):9.
